# Impact of Roasted Yellow Split Pea Flour on Dough Rheology and Quality of Fortified Wheat Breads

**DOI:** 10.3390/foods10081832

**Published:** 2021-08-07

**Authors:** Kali Kotsiou, Dimitrios-Diogenis Sacharidis, Anthia Matsakidou, Costas G. Biliaderis, Athina Lazaridou

**Affiliations:** Laboratory of Food Chemistry and Biochemistry, Department of Food Science and Technology, School of Agriculture, Aristotle University of Thessaloniki, 54124 Thessaloniki, Greece; kkotsiou@agro.auth.gr (K.K.); sacharid@agro.auth.gr (D.-D.S.); matsakidou@chem.auth.gr (A.M.); biliader@agro.auth.gr (C.G.B.)

**Keywords:** starch gelatinization, dough mechanical spectra, dough creep recovery testing, bread texture, starch retrogradation, bread staling, sensory evaluation, bread FTIR, *in vitro* starch digestibility

## Abstract

Roasted yellow split pea (YSP) flours were used to substitute wheat flour, at 10–20% (flour basis) in wheat bread formulations. Rheometry showed that roasted YSP flour addition increased elasticity and resistance to deformation and flow of the composite doughs, particularly at 20% substitution; instead, at 10% addition (either raw or roasted YSP flour), there were no effects on dough rheology and bread textural properties. Breads fortified with roasted YSP flour at levels >10% exhibited lower loaf-specific volume and harder crumb compared to control (bread without YSP flour). Moreover, only breads with 20% roasted YSP flour displayed a significantly higher staling extent and rate, compared to control, as assessed by large deformation mechanical testing and calorimetry (starch retrogradation) of crumb preparations. This formulation also showed a large increase in β-sheets and β-turns at the expense of α-helix and random coil conformations in protein secondary structure as assessed by FTIR spectroscopy. Roasting of YSP effectively masked the “beany” and “grass-like” off-flavors of raw YSP flour at 10% substitution. Overall, roasted YSP flour at the 10% level was successfully incorporated into wheat bread formulations without adversely affecting dough rheology, bread texture, and shelf-life, resulting in final products with a pleasant flavor profile.

## 1. Introduction

Wheat is one of the most important crops in terms of global production and use in various food items. More specifically, the increased consumption of wheat-based products has been associated with the adoption of a “western lifestyle” [1]. The whole grain wheat is, in fact, a great source of carbohydrates, digestible or as fibers, and it also contains significant amounts of other important nutrients, such as proteins, rich in methionine and cysteine, and micronutrients, such as minerals and vitamins, which may contribute to a healthy diet. However, the use of whole grain flours in wheat-based products is usually limited. Thus, most of these products are based on white wheat flours that have undergone refining, consisting mainly of wheat endosperm constituents. Refined grains contain approximately 80% less dietary fiber than whole grains; moreover, production of wheat flours with low extraction rates results in substantial losses in essential minerals, vitamins, and phytonutrients [2,3]. White bread, the most common bakery product, is a staple food in many parts of the world [4], being rich in rapidly digestible carbohydrates and resulting in high postprandial blood glucose levels, which are associated with increased risk for insulin resistance and type 2 diabetes [5]. Additionally, cereal proteins are poor in some of the essential amino acids, mostly, in lysine. Therefore, fortification of bread with ingredients of high nutritional value, such as flours from legumes, might contribute to an improvement in public health.

Dry pulses mainly consist of carbohydrates (60–70% on dry weight basis), and they are superior sources of both dietary fibers and lysine-rich proteins [6,7]. A great variation in the dietary fiber content of the raw seeds has been reported, which varies in the range of 4–39%, depending on the type of legume, the cultivar, and agronomic conditions [6,8]; apparently, the insoluble fiber fraction may reach up to 85–93% of total fibers. It is well known that legume starches can contain high amylose levels (~30–45% of total starch), and therefore, legume seeds are being considered as natural sources of resistant starch [9]. Additionally, these seeds contain other forms of non-digestible carbohydrates, compared to typical cereal-based foods, thus contributing to lower starch digestibility and thereby to reduced postprandial glucose and insulin responses, which are desirable for people with diabetes and for body weight management [10,11]. The potential role of pulse consumption in preventing other chronic diseases, such as heart disease and colorectal cancer, is also well recognized [12]. Finally, the protein content of pulses usually ranges between 15–25%, while in species such as faba bean and lupin, it might even reach up to 40%; additionally, when legumes are combined with cereals in a single meal or a food product, protein efficiency is improved as a result of their complementary essential amino acid profiles [6,13].

Considering the potential role of pulses consumption in preventing chronic diseases, and in order to improve the nutritional quality of grain foods, there have been many attempts to incorporate legumes into bread and other widely consumed cereal-based products, such as biscuits, pasta, snacks, etc., with limited success, mostly leading to products of inferior technological and sensory characteristics [14]. Incorporation of legume flours into a wheat flour-based formulation might largely impact on dough rheological characteristics, and therefore, a better understanding of how such a composite protein network is responding would be an important aspect for improvement of dough handling properties and performance. Empirical rheology, such as farinography, extensography, and alveography, and more recently, by means of the Mixolab System, is often employed to assess the influence of flour constituents and additives on dough behavior during breadmaking. In most cases, the inclusion of legume flours in wheat doughs increased water absorption and dough development time, while dough stability and extensibility are reduced [15,16,17,18,19,20,21]. The changes in the measured parameters were more pronounced at high substitution levels (from 10 to 40%), indicating a concentration dependence. These responses have been attributed to the high water absorbing capacity of legume proteins, which limit the amount of water available for the development of the gluten network, the dilution and interruption of the gluten structure [15,16] as well as to possible interactions between gluten and legume proteins. Even though empirical rheology is a very useful analytical framework to characterize dough systems in the bakery industry, fundamental rheology, and more specifically, creep-recovery testing and oscillatory measurements, can provide a more comprehensive insight into the viscoelastic behavior of the legume–wheat composite doughs [21,22]. Dough fundamental rheological parameters expressing dough resistance to flow and to deformation have been previously correlated with the extent of dough rising during proofing and breadmaking performance [23]. 

As expected, the modifications in dough rheological behavior greatly influence bread volume and crumb textural characteristics, which are important indicators of bread quality attributes, from the consumers’ point of view. Legume flour addition at levels higher than 10%, in most cases, adversely affects specific volume and crumb hardness, as it has been observed with the use of various legume flours in breadmaking, such as chickpea [16,24,25], lentils [17,18,25], and faba bean [26]. Other than textural defects, pulse-fortified breads are characterized by “grassy” and “beany” off-flavors, diminishing consumer acceptability and limiting their incorporation into food products, as it has been previously shown for breads supplemented with chickpea flour [16,24,27].

Pretreatment of legumes prior to milling might have an important impact on flavor improvement of the end products; e.g., bread made with roasted peas was found to have a less intense aroma and pulse flavor compared to that made from untreated peas [28]. Volatile compounds, such as pyrazines and alkylated pyrazines, produced during roasting and cooking could be effective in masking the “beany” off-flavor, often linked with aldehydes, alcohols, and sulfur containing constituents present in raw pulses [29], further to volatilization of the latter compounds upon baking.

Finally, bread staling is an important determinant of bakery products shelf-life, since unfavorable changes in texture during storage often precede microbial spoilage. The increase in protein and fiber content in the fortified products might reduce water availability to gluten, but it might also limit the water migration from the crumb to the crust and/or decrease amylopectin retrogradation. For example, an anti-staling effect of lupin protein isolates as evidenced by delaying bread firming during storage for up to 48 h has been reported [30]; this outcome was more pronounced when a lupin protein isolate was combined with supplementary gluten, indicating a kind of synergistic effect.

Additional research is needed to understand the physicochemical properties of legume flours, in order to promote their incorporation into different types of baked items, especially in bread with improved nutritional attributes, but also with acceptable sensorial characteristics and adequate shelf-life. In the present study, roasting of yellow split pea seed before milling was explored as a quick and cost-effective process for eliminating the undesirable legume off-flavors in the final product and thereby creating products with acceptable sensorial attributes to the consumers. Roasted yellow split pea, usually containing 25–30% of protein, was chosen for partial substitution of wheat flour in bread formulation due to its high nutritional value, aiming at providing a comprehensive investigation of its effect on dough thermal and rheological properties as well as physicochemical, sensory, and nutritional characteristics of the end-product, including the staling events. To our knowledge, for the first time, a multi-instrumental analytical approach including calorimetry, FTIR spectroscopy, and texture analysis was employed for the investigation of quality parameters of both fresh and staled products in an attempt to shed some light on the mechanisms of staling kinetics of pulse flour-supplemented wheat doughs and bread; this approach was also combined with an assessment of the potential nutritional benefits and sensorial attributes, which are important complementary aspects of product acceptance by consumers, besides the shelf-life.

## 2. Materials and Methods

### 2.1. Raw Materials

Wheat flour (WF) with extraction rate of 55% (Type 55), and 11 and 0.70% protein and ash content, respectively, was gifted by Flourmills Thrakis I. Ouzounopoulos, SA (Alexandroupoli, Greece), whereas yellow split pea (Agrino, Greece) and dry instant yeast (Mac Magic from Alimentaria S.A., Greece) were purchased from the local market. 

### 2.2. Roasting and Milling of Yellow Split Pea and Particle Size Distribution of the Obtained Flours

Yellow split peas (YSP) were roasted in a dry nut roaster (Mikropoulos and Co. Ε.Ε., Thessaloniki, Greece) at 150 °C for 25 min in 3 batches of 5 kg each, under continuous mixing to ensure uniform thermal treatment, and then, the roasted YPS seeds from all batches were combined; the color and moisture content of the roasted legumes were determined as described below, and the obtained values from each batch were used as indicators to ensure the repeatability of the roasting treatment. Raw and roasted yellow split pea were milled into flour using a household stone mill (Waldner Combi-Star Grain Mill and Flaker, Lienz, Austria), stored at −18 °C until used and designated as yellow split pea flour (YSPF) and roasted yellow split pea flour (RYSPF), respectively. Particle size distribution of the obtained flours and of WF was determined by sieve analysis, according to Sereti et al. [31], using 200 g of a weighed sample, which was passed through a series of sieves with pore sizes from top to bottom: 500, 250, 150, and 75 μm. The YSPF, RYSPF, and WF exhibited d_50_ (median diameter) of 220, 230, and 95 μm, respectively (Figure S1).

### 2.3. Dough Preparation and Bread Making

The RYSPF preparation was incorporated into breads at 10, 15, and 20% substitution levels of the wheat flour. The following flour samples were used for breadmaking (Table 1): CON (100% WF), YSP10 (mixture of 10% YSPF and 90% WF), RYSP10 (mixture of 10% RYSPF and 90% WF), RYSP15 (mixture of 15% RYSPF and 85% WF), and RYSP20 (mixture of 20% RYSPF and 80% WF); all compositions are expressed on flour basis. For dough preparation, the level of added water to the dough was optimized for each mixture based on water absorption measurements made by farinography. After pre-mixing the flour with 1% dry bakers’ yeast and 2% salt (flour basis) for 5 min to homogenize the solid ingredients, water was added and the dough was kneaded for 40 min in a professional spiral mixer (Resto Italia SK 10 MO, Urbino, Italy). After kneading, the dough was left to rest for 20 min at room temperature, divided and rounded into 280 g individual pieces, and left to rest for another 10 min; subsequently, the dough spherical pieces were molded into loaves, placed into pans, proofed (38–40 °C × 35 min, 75% RH), and finally, baked at 180 °C for 28 min (air-o-stream combi oven, Electrolux Professional SpA, Pordenone, Italy). The breadmaking procedure for each bread formulation was repeated in triplicate.

### 2.4. Empirical Dough Rheological Parameters 

Farinographs obtained by Promylograph Egger T6 (Labortechnik Egger, Neumarkt, Germany) were used to determine the optimum water level of the different dough formulations following the ICC method [32]; other than the water absorption parameter, development time and stability of the dough samples were also evaluated. Promylograph Egger TS6 CE (Labortechnik Egger, Neumarkt, Germany) was used to obtain dough extensographs performed according to the ICC method [33]. The following parameters were calculated: the area under the curve, which expresses the energy required to stretch the dough up to its rupture point, the resistance to extension corresponding to the height of the curve at 50 mm from the beginning of stretching (R50), the dough extensibility (E), which represents the length of curve from the beginning of stretching up to rupture and the ratio of R50/E. All parameters were measured at 45, 90, and 135 min total time as follows: the dough was placed in the humidified chamber of the instrument for resting, and after 45 min, it was stretched up to rupture, it was moved from the holder, reshaped and placed in the chamber for another 45 min, and then, stretched again; this process was repeated one more time (i.e., 135 min total time). The doughs for each rheological measurement were prepared without yeast addition and tested in triplicate.

### 2.5. Fundamental Dough Rheological Properties 

Dough rheological properties (fundamental characterization) were also determined with a rotational Physica MCR 300 rheometer (Anton Paar GmbH, Graz, Austria), using a Paar Physica circulating bath (Graz, Austria) and a controlled peltier system (TEZ 150P/MCR, Graz, Austria) for temperature control. The doughs were prepared as described above, using the same formulations and procedure with breadmaking, but omitting the yeast addition. After mixing, the doughs were wrapped with a plastic membrane to avoid moisture loss and left to rest for 20 min at room temperature, before any rheological measurement. The oscillatory measurements and creep-recovery tests were performed using a parallel plate geometry (50 mm diameter, 2 mm gap), with a solvent trap to avoid moisture loss during measurements. After dough loading on the rheometer, the specimen was left to rest for 15 min prior to any measurement. The temperature was regulated at 25 (±0.1) °C. The doughs for these measurements were tested in triplicate.

Frequency sweep (oscillatory) measurements (mechanical spectra) were performed from 0.1 to 20 Hz under a constant strain (0.1%), which fulfilled the linear viscoelasticity requirements as shown by preliminary strain sweep tests. Storage modulus, G′, loss modulus, G″, complex viscosity, η*, and damping factor, tan δ, (G″/ G′) were recorded. Creep-recovery tests were carried out by applying a constant stress (50 Pa) for 60 s on the dough and allowing strain recovery for 180 s after removal of load. The compliance curve data from the creep-recovery tests were analyzed using the supporting software (US200 V2.21, Anton Paar GmbH, Graz, Austria) of the rheometer and fitted to the Burgers model as described elsewhere [34].

### 2.6. Compositional Analysis of Raw Materials and Breads

The moisture of raw materials and final products (bread crumb and crust) and protein content were determined according to the American Association of Cereal Chemists International official methods 44-15.02 [35] and 46-30.01 [36], respectively.

Total dietary fiber assays were carried out based on the AACC method 32-05 [37] and the AOAC Method 985.29 [38], using the thermostable α-amylase, protease, and amyloglucosidase of Total Dietary Fiber Assay kit of Megazyme (Megazyme International Ireland Ltd., Co. Wicklow, Ireland). For separation of soluble from insoluble dietary fibers, the FibreBags filtration system, which was a gift from Gerhardt Analytical Systems (Königswinter, Germany), was used. The sample (1 g) was subjected to enzymatic digestion with α-amylase, protease, and amyloglucosidase. For insoluble dietary fibers, the sample was filtered, and the residue was washed, dried, and weighed. For the soluble dietary fiber portion, the filtrate was treated with 95% ethanol to precipitate the soluble fibers, filtered, and the residue was weighted. Each sample digestion was carried out, simultaneously, in duplicate, so that one residue from each type of fiber was used for protein determination by the Kjeldahl method using a Gerhardt analytical apparatus, and the other one for ash determination by incineration at 525 °C for 5 h using a muffle furnace (L 9/11/B180 L-090H1CN, Νabertherm GmbH, Lilienthal/Bremen, Germany). Moisture, protein, and total dietary fiber analysis were carried out on both raw materials and breads in triplicate.

Digestible and resistant starch of the breads was determined using the respective assay procedure of Megazyme (K-DSTRS), which is based on a modified method of Englyst et al. [39]. Accordingly, the sample was incubated by a mixture of pancreatic α-amylase and amyloglucosidase at 37 °C for up to 4 h, and aliquots of the digest were removed at 20, 120 and 240 min for estimation of rapidly digested starch (RDS, digested up to 20 min), slowly digestible starch (SDS, digested from 20 to 120 min), and total digestible starch (TDS, digested up to 240 min); the remaining starch after 240 min digestion was defined as resistant starch (RS). The RDS, SDS, and TDS as well as RS followed dissolution in sodium hydroxide were assessed using the Megazyme Glucose Determination Reagent (glucose oxidase/peroxidase; GOPOD) after incubation of the digest fractions with amyloglucosidase to hydrolyze the remaining maltose to glucose. Lyophilized bread samples (in triplicate) were used for the determination of digestible and resistant starch. 

### 2.7. Color Parameters

Color parameters (L*, a*, and b* values of CIE system) of both bread crumb and crust were measured using a Chromameter (Konica Minolta, CR-400 Series, Tokyo, Japan) calibrated with a white tile (L* = 96.9, a* = −0.04, b* = 1.84). Additionally, the hue angle (h_ab_) and Chroma (C*_ab_) parameters were calculated as described elsewhere [40,41]. For both crust and crumb color, three bread samples were used from each breadmaking batch, and five measurements on each sample were averaged into one replicate.

### 2.8. Specific Volume

The specific volume of the loaves was determined using the benchtop laser-based scanner VolScan Profiler VSP600 (Stable Micro Systems, Godalming, UK). Three bread samples were measured from each breadmaking batch.

### 2.9. Textural Attributes

A texture Analyzer TA.XT plus from Stable Micro Systems (Godalming, Surrey, UK) was used for crumb and crust texture analysis. For crumb analysis, a circular shape cutter was employed to obtain cylindrical bread crumb specimens (40 mm diameter × 30 mm height). The samples were submitted to texture profile analysis (TPA) using a 75 mm diameter plate probe. The test was performed at 60% applied deformation, 0.8 mm/s test speed, and 5 s delay time between first and second compression cycles. The TPA parameters, namely, hardness, cohesiveness, springiness, resilience, and chewiness, were calculated according to Armero and Collar [42]. A puncture test was instead used to evaluate crust texture, using a 2.5 mm radius spherical probe at a test speed of 1 mm/s. The probe was applied on a 40 mm × 30 mm piece of the upper crust after removing any residual crumb. The peak force of the force-time curve represented crust hardness. Both tests were performed on fresh baked breads (2 h after baking) and on 1st, 2nd, and 4th day of bread storage at 25 °C, in order to evaluate the staling process. For each test and each time interval, three bread samples were used from each breadmaking batch, while two specimens from each bread were tested, and the obtained values were averaged.

### 2.10. Starch Gelatinization and Retrogradation 

Differential scanning calorimetry (DSC) was performed using the DSC 3 calorimeter (Mettler-Toledo GmbH, Analytical, Zurich, Switzerland). For starch gelatinization, about 12 mg of flour samples were mixed with distilled water (flour: water 30:70), and the slurries were hermetically sealed into DSC stainless steel crucibles and conditioned (proper hydration of flour particles) for 2 h at room temperature; then, the crucibles were heated from 25 to 120 °C (heating rate 5 °C/min). The onset temperature (T_o_), the peak temperature (T_p_), and the melting enthalpy (ΔH_gel_) of starch gelatinization (endothermic transition) were determined from the respective thermograms. For estimation of starch retrogradation, samples of about 12 mg of lyophilized crumb from breads stored for 0, 1, 2, and 4 days at 25 °C were mixed with distilled water (flour: water 30:70) and conditioned as described above. Then, the crucibles were heated from 5 to 120 °C (heating rate 5 °C/min). The onset temperature (T_o_^ret^), the peak temperature (T_p_^ret^), and the melting enthalpy (ΔH_ret_) of the retrograded amylopectin were determined from the respective thermograms (endothermic transition). Moreover, the retrogradation index (RI), which represents the percentage of retrograded amylopectin in relation to that of granular starch undergoing gelatinization, was calculated according to Correa and Ferrero [43] as follows Equation (1):

RI % = (ΔH_ret_ × 100)/ΔH_gel_ (1)

Three replicates were performed for each examined sample.

### 2.11. Fourier Transform Infrared (FTIR) Spectroscopy Analysis

Fresh bread samples (2 h after baking) and those obtained from breads following storage for 1, 2, and 4 days were freeze-dried and used for FTIR analysis to evaluate the effect of storage on protein secondary structure and starch chain reordering. Wheat and raw and roasted YSP flours, as well as freeze-dried crumb of all bread samples, were used to obtain their absorption spectra (32 scans) by a FTIR spectrometer (FTIR 6700 series, JASCO, Tokyo, Japan) at a resolution of 4.0 cm^−1^, in the area of 4000–650 cm^−1^. The samples were placed on an ATR sampling accessory MIRacle ™-Universal ATR (Pike Technologies, Madison, WI, USA) with a 3-Reflection Diamond/ZnSe Performance Crystal Plate, and to attain good contact between flour particles and cell surface, a constant pressure was applied by the pressure tool. Spectra were obtained in triplicate. To avoid significant interference to the spectra signal due to the water high FTIR signal, freeze-dried bread samples were instead used for the FTIR analyses [44]. The CO_2_, H_2_O, and ATR corrections were performed in this order with the aid of Spectramanager v.2.15.15, JASCO. The corrected spectra were then subjected to Savitsky–Golay smoothing (interval 10, polynomial order 3) and baseline correction (adaptive, coarseness 15%) by the software SpectraGryph v.1.2.13 (F. Menges Spectragryph–optical spectroscopy software, Oberstdorf, Germany). The Amide I region between 1580 and 1700 cm^−1^ [45] was further analyzed by applying the second derivative deconvolution procedure, to specify the separate conformations of the protein secondary structures. Curve fitting was performed by MagicPlotStudent v.2.9.3 free software (Magicplot Systems, LLC, Saint Petersburg, Russia). The center positions of the fitted Gaussian curves were specified according to the minima points obtained by the second derivative deconvolution procedure. The fitting process was considered successful when the correlation was better than 0.995. The percentage contribution of each secondary structure obtained was calculated as the ratio of the relative areas under the Gaussian curves by the sum of the fitted model. Measurements were performed in triplicate. The peaks centered in the region at 1620–1644 and in the region at 1690–1695 cm^−1^ have been assigned to the β-sheet structure, peaks centered in the region at 1660–1685 to β-turn structure, peaks centered in the region at 1652–1660 cm^−1^ to α-helix conformation, and peaks centered in the region of 1644–1652 cm^−1^ to random coil structures [44,45,46]. The FTIR analyses were performed in triplicate.

### 2.12. In Vitro Starch Digestibility

*In vitro* starch enzymatic digestibility of the bread crumb was assessed according to Lazaridou et al. [47]. Briefly, 5 g of bread samples were crumbed (using a food processor) to a size of approximately 0.5 cm^3^ and placed in 35 mL of sodium phosphate buffer (20 mM, pH 6.9). The pH of the suspension was adjusted to 1.5 (with HCl) and first digested with pepsin (Sigma-Aldrich, Pool, UK) (575 units/g starch) for 30 min at 37 °C. The pH of the mixture was subsequently re-adjusted to pH 6.9 (with NaOH), the volume of the liquid was made up to 50 mL with a solution of sodium phosphate buffer (20 mM, pH 6.9), and porcine pancreatic α-amylase (Megazyme) (110 units/g starch) was added. The suspension was transferred to a dialysis tube (cellulose membranes, MW cut-off 14,000, Sigma-Aldrich, St. Louis, MO, USA), which was placed in a screw cap glass bottle containing 450 mL of sodium phosphate buffer (20 mM, pH 6.9) for 5 h at 37 °C under mild stirring in a water bath shaker (Memmert WNB 7-45, Memmert GmbH + Co. KG, Buchenbach, Germany). Aliquots (1 mL) of dialysates were taken at 20, 60, 120, 180, 240, and 300 min in duplicate. The aliquots were incubated with amyloglucosidase (from Rhizopus mold, Sigma-Aldrich), and the released glucose was measured using the GOPOD reagent. For evaluation of *in vitro* starch digestibility, the area under the curve (AUC) of released glucose over 300 min of digestion was calculated. The analysis was performed in triplicate.

### 2.13. Sensory Analysis

A preliminary sensory analysis was carried out at the beginning of the present study to figure out whether the sensory characteristics of the fortified breads with flour from raw or roasted YSP were acceptable by the panel. Thus, 20 individuals were selected to perform a paired preference test in order to choose the most preferable sample between the fortified with 10% raw YSP flour and that with 10% roasted YSP flour. The assessors were selected as potential consumers of the product, based on their responses in a previously distributed questionnaire, indicating that they daily consume wheat bread. Approximately 75% (data not given) of the assessors preferred the formulation with the addition of 10% roasted YSP flour, and therefore, this type of flour was chosen for studying its impact on sensory characteristics of the fortified products at higher levels of supplementation (up to 20%). 

Quantitative descriptive analysis of the final products was carried out by a trained panel of 18 assessors, focusing on flavor characteristics, such as “wheat bread”, beany, green (grass-like), earthy, roasted, and over-roasted (burnt). The process included several meetings in order first to define the perceived flavors and followed by training to familiarize the panelists with the sought flavor notes. 

### 2.14. Statistical Analysis

The mean values of all analyzed parameters were compared using Tukey’s test at α = 0.05 significance level.

## 3. Results and Discussions

### 3.1. Starch Gelatinization Properties of Yellow Split Pea Fortified Wheat-Based Flours

The endothermic peak of starch gelatinization of wheat flour (Τ_p_) was at 63.76 °C (Table 1, Figure S2), which is typical for this matrix. Legume flours (raw and roasted) were characterized by significantly higher (*p* < 0.05) onset (T_o_) and peak (T_p_) gelatinization temperatures, compared to wheat flour alone. Higher gelatinization temperatures have been previously found for starch of different legume species than that of cereal species, attributed to the higher amylose content of starch, i.e., ~30–40% for the former than ~20–25% for the latter [9]. On the other hand, the apparent gelatinization enthalpy (ΔH_gel_) of YSPF or RYSPF was not significantly different compared to that of WF (Table 1). Additionally, the inclusion of up to 20% legume flours in the wheat flour did not have any significant effect on any of the gelatinization properties of the flour mixture, which indicates that these levels of substitution did not significantly affect starch thermal properties of the composite flours. In a recent study, the addition of 30% broad beans flour in a wheat dough resulted in a shift of T_p_ to higher values, which might originate from the higher level of legume flour substitution and the different type of starch present in these preparations [48].

### 3.2. Empirical Rheological Parameters of Yellow Split Pea Fortified Wheat-Based Doughs 

Parameters derived from farinograph are presented in Table 2. Addition of 10% flour from either raw or roasted YSP flour did not have any negative effect on dough development time and stability of the doughs. At higher levels of yellow pea flour inclusion (15 and 20%), significant increases in the development time and decreased stability of the dough were noted; this behavior is probably related to the dilution and interruption of gluten network continuity and/or gluten–legume protein interactions. In previous studies, significantly longer development times and decreased stabilities were also observed with the use of flours of raw and germinated pea at levels up to 15% [49], raw, germinated, and toasted yellow pea at 30% [50], raw and germinated yellow pea and faba bean at 10–20% [21], and raw and steam-processed pea and split pea at a level of 20% [19].

Regarding the extensograph parameters after 45 min of resting time, substitution of wheat flour with 10% raw YSP flour significantly increased the stretch energy, meaning that a larger amount of work is required for dough deformation, compared to CON sample, while roasting seemed to decrease this parameter; as a result, RYSP20 showed significantly lower stretch energy and extensibility than CON (Table 2). Moreover, all composite doughs, after 45 min of resting time, exhibited R50 and R50/E ratios similar to the wheat dough. The latter observation is very important, since R50/E values are correlated to bread dough quality and baking performance [51] as well as to some fundamental rheological parameters (tan δ) as determined by dynamic rheometry measurements [52]. In fact, a balanced ratio between the resistance to extension and extensibility is related to dough expansion and gas holding capability. Furthermore, all composite doughs, containing roasted YSP, were able to recover their extensograph characteristics upon mechanical handling and resting (three cycles), indicating that these mixtures are suitable for bread recipes that require multiple cycles of kneading and resting (Table 2). A previous study showed that addition of raw chickpea flour at levels up to 30% decreased the stretch energy and R50, with increasing substitution level, which was attributed to the presence of enzymes or constituents that interact with gluten proteins and inhibit the development of desirable rheological properties for the composite dough network system [16]. 

### 3.3. Fundamental Rheological Parameters of Yellow Split Pea Fortified Wheat-Based Doughs

**Figure 1:**
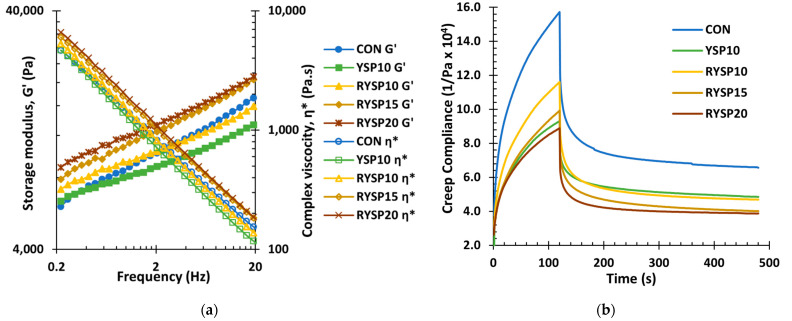
Representative curves of frequency sweep (**a**) and creep recovery test (**b**) of wheat doughs fortified with yellow split pea flour; notation of samples as in Table 1.

As expected, frequency sweep tests confirmed the elastic-like behavior of the tested wheat-based dough formulations, since the storage modulus (G′) was higher than the loss modulus (G″), while both moduli slightly increased and complex viscosity (η*) sharply decreased with frequency (Figure 1a). As shown in Table 3, for a selected frequency of 5.37 Hz, storage and loss moduli and complex viscosity shifted towards higher values, with an increasing level of roasted legume flour incorporated into the composite dough. Even though the values of the examined rheological parameters among the fortified systems did not show any significant difference compared to CON, the RYSP20 was characterized by significantly higher values compared to YSP10, which indicates the formation of a more elastic and firm dough. A structural modification of the YSP proteins due to roasting as well as moisture redistribution among the constituents of the composite doughs might enhance the gel network structure of the system. According to previous studies, the observed differences in the fortified doughs depend on the type and pre-processing of the flour used, as well as the fortification level. Specifically, the addition of 20% raw or germinated yellow pea and faba bean flours resulted in doughs with significantly higher G′ and G″ at the frequency of 10 Hz [21]. Ahmed et al. [22] also reported a concentration dependent effect, since the G′ and G′′ values increased as the concentration of lupine fiber in the composite doughs increased from 5 to 15%. In another study, addition of 30% flours from peas that had undergone different pre-treatments showed that doughs with germinated and toasted flours had significantly higher G′, G′′, and η*, whereas the dough with the raw pea did not differ from the control [50]; those findings are consistent with these of the present work (Table 3).

Regarding the creep-recovery test, the obtained rheological responses were typical of viscoelastic behavior, as expected for wheat-based doughs, showing the creep compliance data to slightly decrease with increasing roasted legume flour level (Figure 1b). All rheological parameters calculated by fitting the creep-recovery test data to the Burgers model are summarized in Table 3. The RYSP10 showed similar rheological properties with the CON sample, in both creep and recovery phases, whereas the YSP10, RYSP15, and RYSP20 exhibited significantly (*p* < 0.05) lower maximum creep compliance (J_max_) and viscoelastic compliance (J_m_) in the creep phase. The YSP10 and RYSP20 were also characterized by significantly lower maximum creep strain compared to control. The RYSP20 dough showed the highest zero shear viscosity (η_o_), which along with the lowest J_max_, describes a dough with a greater resistance to flow and deformation, in agreement with the highest η* and G’ values of this sample, respectively. In previous studies, shifting of the maximum creep strain to lower values and η_o_ to higher values have been related to stronger doughs [23]. It is worth noting here that the more elastic and viscous dough enhances the dough stiffness and is expected to be resistant to expansion upon proofing resulting in breads with low loaf volume. In the recovery phase, no differences were recorded among the samples for most of the measured parameters, apart from the RYSP20 sample, which exhibited significantly lower viscoelastic compliance (J_m_) compared to control. The rheological properties of dough inevitably affect the textural characteristics of the final product, influencing bread quality, since the dough must be sufficiently elastic to allow the formation of the three-dimensional deformable network and strong enough to maintain the bubble structures formed during proofing and baking. Lupin fiber inclusion in a wheat dough has been found to exhibit a similar pattern, since at increased concentration (15% on flour basis), there was a significant decrease in maximum deformation, compared to lower levels of substitution [22]. Overall, substitutions at 5 to 10% result in composite doughs with rheological properties similar to those of wheat flour doughs [16,17], while at higher substitution levels, with addition of vital wheat gluten, there is restoration of the rheological properties, as observed in the case of lentil-wheat composite doughs [17].

### 3.4. Appearance of Yellow Split Pea Fortified Wheat-Based Breads 

**Figure 2:**
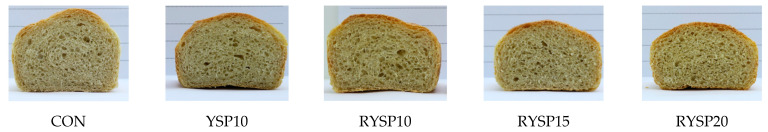
Images of center slices of wheat flour–yellow split pea composite breads; notation of samples as in Table 1.

The appearance of the fortified wheat breads with yellow split pea flours was evaluated by loaf-specific volume measurements and crust and crumb color parameters. Loaf-specific volume was negatively affected by the higher level of legume flour addition, as the RYSP15 and RYSP20 exhibited significantly lower specific volume values than the CON sample (Table 4 and Figure 2). Additionally, the reduced loaf volume of the fortified breads with high levels of YSP flour was associated with a more compact macrostructure of the crumb (Figure 2). Probably a stronger solid-like character and increased resistance to flow and deformation of the fortified doughs (Table 3) led to increased stiffness, resistance to expansion, and ability to regain their initial shape and thereby caused reduced loaf volumes. The dilution of gluten network by incorporation of a non-wheat flour at high levels could also contribute to the decreased loaf volumes of the fortified breads. Similarly, in previous studies, the addition of legume flours from chickpeas, lentil, faba bean, carob bean, etc., at levels higher than 10% (on wheat flour basis), in most cases significantly reduced the loaf volume, regardless the type of the legume employed [16,18,24,25,26]. However, at higher levels of legume inclusion, the addition of vital wheat gluten can improve the specific volume of the produced loaves, by restoring the rheological properties of the dough, as it has been shown with mixtures containing 20% lentil flour [17], or 30% flour from chickpeas, lentils, peas, and soybeans [25].

The crust and crumb color parameters of the examined breads are summarized in Table 4. Inclusion of YSP flours in the formulations gradually reduced bread crust lightness (L*), with RYSP15 and RYSP20 being significantly less bright compared to CON. Moreover, an increase in redness (a*) was recorded in all composite breads, and at the same time, YSP10, RYSP15, and RYSPP20 exhibited significantly lower b* values (yellowness). In this context, there was a reduction in hue angle and Chroma values in crust with increasing level of yellow split pea flour substitution in the bread formulations; the former implies a shift to a redder and less yellow hue and the latter a change from a more saturated color (brighter) to a more achromatic (closer to grey) color. Regarding crumb color parameters, differences were noted only in the case of RYSP20, which showed significantly less bright (lower L* value), redder (higher a*), and yellow (higher b*) values than CON. More distinct differences in color parameters were noted in the bread crust samples, since they are attributed not only to the different color of legume flours added, but also to Maillard and caramelization reaction products, since the surface temperature during baking reaches ~180 °C, whereas the crumb temperature usually does not exceed 96 °C; furthermore, at the end of baking, the crust water activity reaches an intermediate value, ~0.75, which favors the development of Maillard reaction products compared to the high a_w_ values in crumb, ~0.97 [40]. Similar results have been obtained for breads fortified with 20% flour from whole yellow pea that had been roasted at several temperatures, since, in all cases, crumb L* values were lower, whereas a* and b* values were higher than those of control sample [28]. Consumers are not negatively affected by red and especially yellow color shades of breads, probably because they are related with familiar bakery ingredients/flours, such as red wheat, corn, etc.; i.e., despite the significant instrumentally observed color difference towards yellow, for pan breads fortified with thermally treated yellow split pea, the acceptability by the consumers did not appear to be affected [53]. 

### 3.5. Textural Characteristics and Staling Kinetics of Yellow Split Pea Fortified Wheat-Based Breads

**Figure 3:**
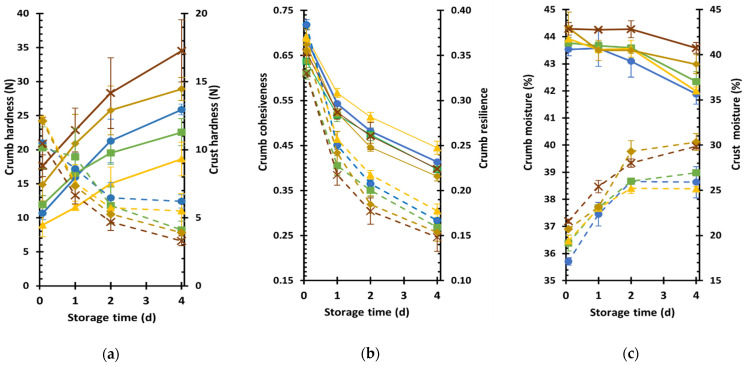
Kinetics of crumb (**—**) and crust (**– –**) hardness (**a**), crumb cohesiveness (**—**) and resilience (**– –**) (**b**), and crumb (**—**) and crust (**– –**) moisture (**c**) of wheat breads fortified with yellow split pea flours: CON (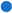), YSP10 (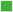), RYSP10 (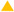), RYSP15 (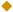), RYSP20 (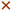); notation of samples as in Table 1.

The textural characteristics of bread samples and their staling kinetics, as assessed by changes in crumb and crust textural attributes during product storage at 25 °C, are presented in Table 5 and Figure 3. The RYSP10 product exhibited the softest crumb throughout storage among the tested samples as indicated by the significantly lower hardness value, compared to those of RYSP15 and RYSP20, which had the higher hardness values among all samples (Table 5, Figure 3a). Additionally, the RYSP20 exhibited a significantly higher (*p* < 0.05) hardening rate compared to YSP10 and RYSP10, indicating a quicker staling process. The increased crumb firmness of breads with the highest yellow split pea level is consistent with its reduced loaf volumes and rather compact crumb structure (Table 4, Figure 2); significant negative correlations between loaf volume of breads and crumb hardness evaluated by compression testing have been previously found [54,55]. In a relevant study, lentil–wheat composite breads exhibited harder crumb texture than the control sample, even at 5% inclusion level, although it has been proposed that a simultaneous use of gluten in the mixture might allow lentil substitution levels up to 15%, without negatively affecting crumb softness [17].

Cohesiveness and springiness values were similar among all fresh samples, while the RYSP20 crumb was less resilient compared to CON (Table 5). Resilience decreased during storage, as shown in Figure 3b, indicating the loss of elasticity due to the staling process. Fresh RYSP20 loaves also exhibited the highest chewiness among all tested bread formulations, although during staling, its chewiness increase rate was the lowest among samples (Table 5). The high level of legume flour inclusion in RYSP20 formulation, which can lead to a substantial decrease in gluten concentration and rather large interference in the development of a well-structured wheat protein network by the proteins and dietary fibers of yellow split pea, might have contributed to the formation of a harder and less elastic crumb.

Overall, the staling process during storage negatively affects bread texture resulting in harder, less elastic, and crumblier crumb (Figure 3a,b); the latter is evident by the decrease in crumb cohesiveness values with increasing storage time. Staling is a complex phenomenon attributed to several mechanisms that occur in bakery items; the most important ones being gluten dehydration, leading to the transition of gluten from a rubbery to a glassy state [56], amylopectin retrogradation, and water transfer from crumb to crust [57]. Finally, all fresh samples exhibited similar crust hardness levels (Table 5), indicating that the applied substitution levels did not have any substantial effect on this parameter. Nevertheless, crust softening upon bread storage (Figure 3a) due to staling occurred at a higher rate for bread containing raw YSP than the control, while the inclusion of roasted YPS into the bread formulation also appeared to increase the crust softening rate, but not in a concentration-dependent manner (Table 5). It is worthy to note that findings of the present study are referred to the samples prepared under the conditions described in this work and for the specific commercial yellow split pea used. There are several factors that could affect the quality characteristics of bakery products supplemented with legume seeds, such as variety, growth location, storage time [58], after harvest, and after milling of the seeds as well as flour particle size [40,47].

Crust softening during staling was also evidenced (Figure 3c) and is generally ascribed to water redistribution between crust and crumb. In fresh breads, crumb moisture content did not differ among the samples, while an increase in crust moisture with an increasing level of YSP flour into the bread formulation was noted probably due to the increasing content of proteins and dietary fibers that enhance water retention (Table 5); at the end of storage, the fortified breads with RYSP15 and RYSP20 had considerably higher crust moisture compared to control sample (Figure 3c). Instead, fortification of breads with YSP at any level did not seem to have an impact on moisture gain rate of crust, whereas the moisture loss rate of crumb decreased significantly compared to control bread, only in case of the highest fortification level (RYSP20) (Table 5), resulting in a product with the highest moisture content at the end of storage (Figure 3c).

### 3.6. Amylopectin Retrogradation Kinetics of Yellow Split Pea Fortified Wheat-Based Breads

**Figure 4:**
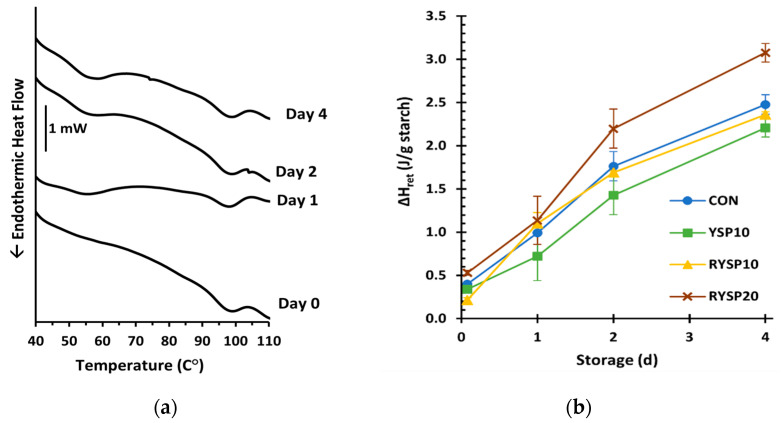
Representative DSC thermograms of RYSP10 sample during staling (**a**) and kinetics of starch retrogradation apparent melting enthalpy (ΔH_ret_) (**b**) of wheat breads fortified with yellow split pea flours and stored at 25 °C; notation of samples as in Table 1.

The retrogradation of amylopectin in bread crumb during storage was monitored using DSC (Table 6, Figure 4). The onset (T_o_^ret^) and peak (T_p_^ret^) retrogradation temperatures did not show any differences among the tested samples, probably because the substitution levels were relatively low to have an impact. The ΔH_ret_, corresponding to the melting enthalpy of retrograded amylopectin, reflects the extent of retrogradation upon storage (Figure 4). The RYSP20 exhibited the highest rate of amylopectin retrogradation (Table 6, Figure 4b), which might be attributed to the lower water loss rate (Table 5) and, thus, a higher moisture content of bread during storage (Figure 3c) that promotes amylopectin retrogradation [59,60]. Moreover, starches with higher amylose content, such as in legumes, are known to exhibit higher retrogradation rates [61]. Finally, the calculated retrogradation index (RI), which represents the relation between retrogradation and gelatinization enthalpies also suggested that starch retrograded to a higher extent in the RYSP20 formulation; an RI of 43.3 % in comparison to the other tested samples with RI of ~ 35% (Table 6). The highest degree of amylopectin retrogradation of RYSP20 among all bread formulations, as determined with calorimetry, seems to corroborate the highest value of crumb hardness for this sample as measured by TPA test (Figure 3a), implying that these two phenomena are strongly interrelated.

### 3.7. FTIR Spectroscopy Analysis of Yellow Split Pea Fortified Wheat-Based Flours and Breads

**Figure 5:**
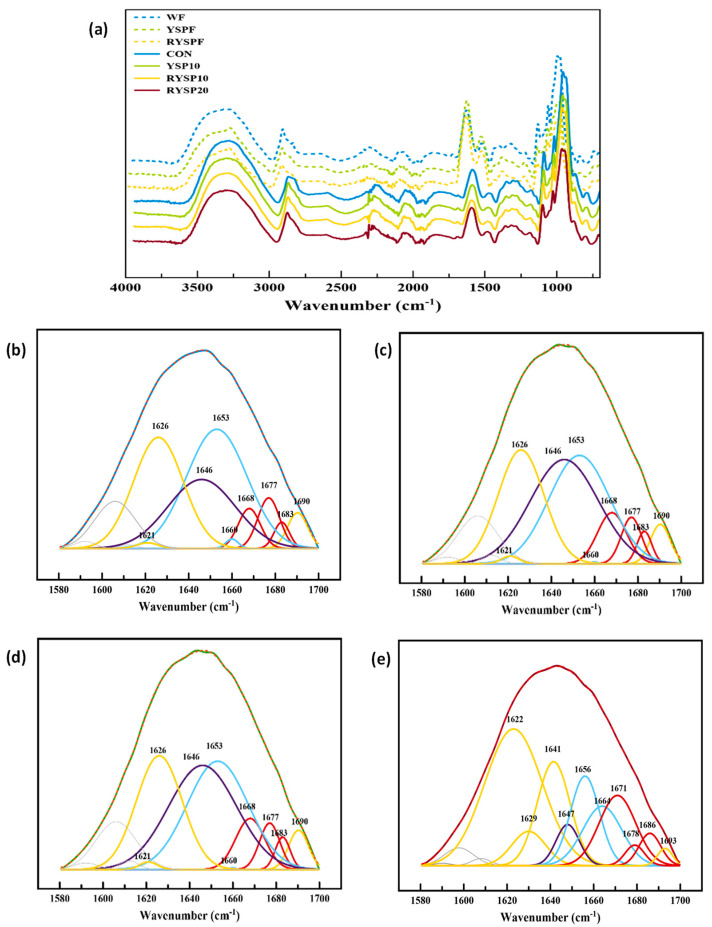
Normalized FTIR spectra of wheat flour (WF), raw (YSPF), and roasted (RYSPF) yellow split pea flours and crumb of the fresh breads (CON, YSP10, RYSP10, RYSPF20) (**a**) and the peak fitted area of the deconvoluted by second derivative Amide I area of CON (**b**), YSP10 (**c**), RYSP10 (**d**), and RYSP20 (**e**); dotted line represents the fitted curve, while the solid line is the original spectrum. Notation of samples as in Table 1.

Figure 5a presents the FTIR normalized full spectra of wheat and raw or roasted yellow split pea flours and crumb samples of fresh breads. There are three distinct regions in all spectra. The bands around 2800–3000 cm^−1^ are attributed to the C-H stretching modes, and the broad band at ~3300 cm^−1^ corresponds to intermolecular H-bonding (O-H stretching modes) [44]. As was expected, the broad peak in the region of -OH vibrations was less intense in the case of roasted YSP flour, probably, as a response to the loss of water due to the roasting process of the YSP flour (Figure 5a); RYSPF flour had 8.3% moisture content versus 10.3% of the YSPF. The region at 1700–1500 cm^−1^ is characteristic of the presence of protein molecules and is attributed to the Amide I (80% C = O stretch, 10% C-N stretch) and Amide II (60%N-H bend, 30%C-N stretch, and 10% C-C stretch) bond vibrations [44,62]. The higher protein content of YSPF (27.0%) and RYSPF (27.5%) flours compared to wheat flour alone (11.1%) was reflected in the higher intensity of the bands in this region observed for the legume flour samples (Figure 5a). 

Deconvolution of the Amide I peak of the flour spectra yielded several peaks attributed to different secondary structures of the protein components (Figure 5b–e). CON, YSP10, and RYSP10 Amide I band deconvoluted to peaks centered at 1621, 1626, and 1690 cm^−1^, assigned to the β-sheet structure, peaks centered at 1668, 1677, and 1683 cm^−1^, assigned to the β-turn structure, peaks centered at 1653 and 1660 cm^−1^, assigned to α-helix structure, and a peak centered at 1646 cm^−1^, assigned to random coil structures. The RYSP20 sample revealed peaks centered at 1622, 1629, and 1693 cm^−1^ for β-sheet structure, peaks centered at 1671, 1678, and 1686 cm^−1^ for β-turn structure, peaks centered at 1656 and 1664 cm^−1^ for α-helix structure, and a peak centered at 1647 cm^−1^ for random coil structure [44,45,46]. The estimated protein secondary structures of the flours are given in Table 7. The protein conformation of the β-sheet structure was the most abundant structure in wheat flour (41.5%), which is in agreement with previously published data [63,64]. Raw and roasted YSP flours, also, had a high content in β-sheet structures, 47.8% and 45.4% for raw and roasted flour, respectively (Table 7). Similarly, it has been reported that pea, lentil, and common bean flour have a high content of β-sheet conformations [63,65]. Roasting of yellow split pea flour led to a small conformational change in the proteins, seemingly, from the β-sheet structure (a decrease from 47.8% to 45.4%) towards random structure (an increase from 13.3% to 15.4%) (Table 7), leading to a more disordered conformation [65,66]. This has been also reported for thermally treated common bean flours and was attributed to protein unfolding and denaturation events [65]. No change for the α-helix of the YSP flour was noted after roasting, which was consistent with the results reported for dry gluten heated up to 85 °C [67] and for thermally treated common bean flour at 120 °C for 24 h [65]. The YSP flours exhibited a much higher content of the random structure and lower α-helices of proteins compared to wheat flour alone (Table 7); other constituents in legume flours might disrupt the hydrogen bonding in some α-helices and destabilize towards random coil structures [68].

Figure 5b–e presents the deconvolution and peak fitting procedure applied to the spectra region of Amide I to obtain the ratios of the secondary structures of proteins in crumb of fresh breads. As it is elucidated by the areas obtained from the Amide I band constituents, control and samples containing raw or roasted YSP at the 10% level exhibited similar secondary structures with almost equally distributed structures between random coil (24.5–28.5%), α-helix (25.2–29.1%) and β-sheet (25.8–27.9%), while the β-turn conformation was the least favored in these samples (10.7–11.7%) (Table 7). However, incorporation of roasted YPS flour at the highest level (20%) into the bread formulation largely affected the protein secondary structure by greatly increasing β-sheets (52.2%) and β-turns (26.0%), at the expense of α-helix (10.9%) and random (3.9%) conformation, i.e., the increase in β-sheets has been associated with dehydration of gluten that results in chain aggregation through intermolecular β-sheets [44,45,69]. The increase in β-sheets could also be partly attributed to legume protein aggregation due to their possible denaturation following the roasting treatment. Concerning gluten aggregation, it seems feasible to occur in the case of RYSP20 bread, since the YSP flour at 20% greatly fortified this formulation with dietary fibers and proteins (Table 8) that may cause dehydration of gluten due to competition for water with these polymers; i.e., gluten dehydration could strengthen the macrostructure of crumb resulting in low volume [70], hardening of the crumb, and fast staling [71], phenomena in agreement with the data presented in Table 5 and Figure 3. In previous studies, it has been found that fortification of dough and bread formulations with flours or flour fractions or concentrates enriched in dietary fibers resulted in water redistribution, which is accompanied by a shift of the secondary structure of gluten from β-spiral (consecutive β-turns) to β-sheet conformation [44,45,69,70]; such a conformational transition has adverse effects on bread quality. In our study, the increase in β-sheets in secondary protein structure of the RYSP20 bread sample is accompanied with an increase in β-turns as well (Table 7). There was also a decrease in α-helices content and a large increase in β-sheets in the protein structures of RYSP20 bread compared to all other samples (Table 7) that could at least in part be attributed to the presence of legume globulins, resulting in poor bread quality.

The evolution of the CON, YSP10, RYSP10, and RYSP20 protein secondary structures was also monitored with FTIR spectra through storage time (Table 7). The protein chains in the crumb of CON, YSP10, and RYSP20 samples reorganized upon storage, as it was elucidated by decrease in α-helix conformation and an increase in random conformation in the case of CON and YSP10 from 0 day (fresh breads) until the end of storage (4th day). The available data about changes in protein secondary structure in bread upon storage are very limited, focusing mostly on the gliadin and glutenin fractions extracted from steamed bread [66]. In the latter study, a downward trend in α-helix and β-turn content in the gliadin and glutenin components was noticed along with an opposite trend regarding β-sheet and random structures, as a result of the loss of moisture upon storage. In the present study, only the RYSP10 sample showed an increase in β-sheets, while the YPS10 and RYSP20 breads surprisingly showed small, but significant (*p* < 0.05) increased estimates of β-turns. As reported by other investigators, for protein structures in a complex system, such as bread, conformational stabilities are very sensitive to interactions with the water molecules and presence of polysaccharides, mainly due to gluten dehydration upon kneading and baking [44,45,72]. Sivam et al. [69], suggested that the presence of other proteins, such as albumins, globulins, prolamins, glutelins, or non-starch polysaccharides, and fibers present in wheat flour should have an impact on the gluten protein network. Since legume flours, in general, contain such proteins, their inclusion in composite cereal flours is expected to further alter the gluten network. Rearrangements of the protein structure during storage of the baked product could be attributed to water molecule redistribution among the various constituents in the composite bread matrix as well as to water loss (Table 5, Figure 3). 

The spectral features of flour and bread samples (Figure 5a) around 1200–800 cm^−1^ are attributed to the carbohydrate region and are associated with starch. The peaks around 1010–1020, 1080, and 1150 cm^−1^ have been attributed to the coupled C-O and C-C stretching vibrations of the polysaccharide molecules [44]. The higher starch content of wheat flour was reflected by the higher intensities of this area, compared to the raw and roasted YSP flours (Figure 5a). The key bands at 1047 and 1022 cm^−1^ have been previously assigned to short-range molecularly ordered or crystalline structures and amorphous forms of starch, respectively [73,74], and therefore, the ratio (R_1047/1022_) of the intensities at 1047 and 1022 cm^−1^ has been adopted as an indicator of the relative level of starch chain ordering [62]. Table 7 presents the estimated R_1047/1022_ values for the flours used in the present study. Crystallinity calculated by this index was not significantly (*p* > 0.05) different between wheat and raw YSP flours being 0.455 for both samples; this value is lower than those reported for wheat and waxy maize starches (0.63 and 0.69, respectively) [75] due to the presence of amorphous amylose in wheat and YSP starches of flours used in the present work. RYSPF displayed significantly (*p* < 0.05) lower R_1047/1022_ ratio (0.432) compared to YSPF probably due to the thermal processing, which may have caused some disordering of organized chains in starch granules. 

Moreover, the ratio R_1047/1022_ has been proposed as a relative index for monitoring starch retrogradation during bread storage and, thus, product staling [62,74], as a result of formation of ordered structures involving starch chains (corresponding to 1047 cm^−1^ band) and the loss of amorphous chain domains (corresponding to 1022 cm^−1^). As expected, the increase in starch molecular ordering and the decrease in the amorphous starch structure, due to retrogradation events, were evidenced by the increased R_1047/1022_ values calculated for the crumb during storage, although such an increase was pronounced only for the RYSP20 bread (Table 7); the latter was consistent with the apparent melting enthalpy values of the retrograded amylopectin, ΔH_ret_, and the retrogradation index, RI, values (Figure 4b, Table 6) of this sample, as assessed from the DSC data. Additionally, the RYSP20 bread exhibited a significantly higher rate of increase for the R_1047/1022_ index during product storage compared to other samples (Table 7). The greater the rate and extent of starch retrogradation during storage of breads fortified with roasted YSP flour at the highest level (20%), as evidenced by both FTIR and DSC analyses, are also consistent with the harder bread crumb (higher hardening rate) when compared to the other tested bread formulations (Figure 3a, Table 5). Similarly, other researchers have reported an increase in the R_1047/1022_ value during the storage of bakery products due to starch retrogradation that also concurred with increased crumb hardness [62,73,76,77,78]. 

### 3.8. Sensory Characteristics of Yellow Split Pea Fortified Wheat-Based Breads 

**Figure 6:**
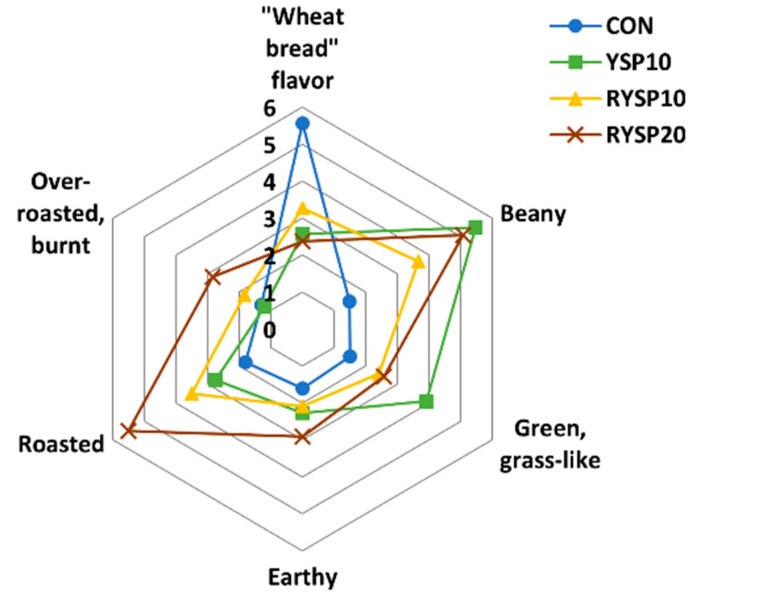
Graphical display of sensory attributes based on quantitative descriptive analysis of composite wheat breads, fortified with yellow split pea flour; notation of samples as in Table 1.

The flavor profile analysis, as presented in Figure 6, showed that samples with YSP flour addition exhibited significantly (*p* < 0.05) reduced “wheat bread” flavor, as that typically recorded for the CON bread preparation. Sample YSP10, which contained the untreated (raw) YSP flour, had higher scores for “green, grass-like” and “beany” flavors, while flour from roasted YSP, when added at 10%, seemed to effectively reduce the aforementioned attributes, and at the same time, a characteristic, pleasant, “roasted” flavor was developed. Inclusion of 20% of flour from YSP increased the “roasted” perception and the “beany flavor” as well, suggesting that the roasting pretreatment of YSP seeds could mask completely, only the “green, grass-like” in the final product when this legume was added to the bread formulations at relatively high levels. Low volume, compact crumb structure, and “beany” and “grass-like” off-flavors are the major defects of legume flour inclusion into bread formulations. 

It has been suggested that the use of gluten or carboxymethylcellulose can significantly improve the volume and crumb characteristics of breads fortified with high levels of legumes, providing breads with accepted textural characteristics [79]. On the other hand, the characteristic off-flavors are very difficult to conceal, since the addition of flavor enhancers is not a common practice in bread formulations. Thermal treatment of legumes prior to milling seems to be quite promising in improving the flavor of the obtained flours. In a recent study, the “pulse flavor” of breads fortified with 20% whole yellow pea flour was significantly reduced, with roasting the seeds prior to milling using the conventional oven method or the Revtech technology, a continuous method that employs direct contact of seeds with the heating spiral [28]. 

### 3.9. Nutritional Quality of Yellow Split Pea Fortified Wheat-Based Breads 

Addition of YSP flours improved the nutritional quality of breads, to a certain degree, depending on the fortification level. Protein content adequately increased, only in the case of RYSP20, while all legume-fortified breads had higher total dietary fiber (TDF) content compared to control (Table 8); all the fortified formulations with YSP could carry the claim of “source of fiber”, according to Regulation (EC) No 1924/2006 of European Union [80], as products containing at least 3 g of fiber per 100 g of product. Soluble fiber was in all cases lower than 10% of the TDF.

Finally, the potential effect of composite breads in reducing the glycemic responses was assessed by glucose release during *in vitro* enzymatic starch digestion of breads. Inclusion of roasted YSP at 20% level into the bread formulation significantly decreased the areas under the curves (AUC), calculated over 300 min of digestion, compared to control bread (Table 8), which could be attributed, at least in part, to the more compact structure of crumb and the resultant low loaf volume of this sample (Figure 2, Table 4), since the total digestible starch did not differ among samples. Reducing the glycemic index of white breads is very challenging because the elevated temperature during baking and the high moisture content of dough favor starch gelatinization, which enhances the enzymic susceptibility digestion of α-D-glucans. In breads with similar composition, their crumb structure is an important factor that influences the glycemic response; it has been previously shown that a more compact crumb structure and/or lower loaf volume in bakery products result in lower *in vitro* and in vivo glycemic responses, due to the reduced accessibility of amylase to the substrate [47,81,82]. However, the increased contents of resistant starch (RS) and dietary fiber in RYSP20 breads could also have an impact on the decreased starch digestibility of this type of products (Table 8). Dietary fibers, both insoluble and soluble, are well known to reduce starch digestibility in starchy products, since they limit starch swelling and gelatinization, and they also restrict both the diffusion of amylolytic enzymes and the following release of starch hydrolysates by acting as physical barriers, adhering on the starch granules in multicomponent food matrices [83].

## 4. Conclusions

Incorporation of roasted yellow split pea (YSP) flour into bread formulations at 10, 15, and 20% substitution level of wheat flour has been investigated in this study. Multi-instrumental analysis of doughs and breads as well as sensory evaluation of the final products were employed for the assessment of dough rheological properties and textural, nutritional, and sensorial attributes of breads as well as for evaluation of bread staling events. Rheometry showed that the addition of flour from roasted YSP at a 20% level exhibited a significant effect on dough rheological parameters by increasing storage modulus and zero shear viscosity as well as decreasing maximum creep compliance, indicating a more elastic dough with a greater resistance to flow and deformation. Parameters derived from farinographs showed that both higher levels (15 and 20%) of legume flour inclusion significantly increased development time and decreased stability of the dough. Moreover, extensographs showed that stretch energy and ratio of resistance to extension/extensibility of dough fortified with 20% roasted legume flour was reduced compared to control formulation made solely by wheat flour, implying a poor baking performance for the composite flours. Inclusion of 10% raw or roasted YSP flour into bread formulations did not have any significant effect on loaf specific volume or on crumb textural properties as evaluated by texture profile analysis. Instead, breads with 15 and 20% roasted YSP exhibited significantly lower specific volume and higher crumb hardness than control, while a product with 20% YSP addition level also had higher chewiness and lower resilience of bread crumb, higher crust moisture content, and lower moisture loss rate of crumb upon bread storage, presumably due to presence of higher dietary fiber and protein concentrations. Therefore, it appeared that the high resistance to deformation and flow of dough fortified with 20% YSP flour resulted in low loaf-specific volume (18% lower than control), which subsequently led to a harder bread crumb and a more compact macrostructure. Moreover, for the latter bread sample, a disruption of the gluten network was revealed by the FTIR analysis of the protein secondary structure, as shown by the large increase in β-sheets in the crumb of this formulation, associated with dehydration of the gluten network, probably due to the presence of high amounts of dietary fiber and legume globulins into the composite dough system; migration of water from gluten to other bread components including starch could also contribute to observed increases in crumb hardening and starch retrogradation rates upon bread storage. Breads formulated with 20% roasted YSP flour exhibited the highest changes in these properties. 

Sensory evaluation using a trained panel revealed that roasting of YSP seeds before milling can effectively reduce the “beany” and “grass-like” off-flavors often detected for the fortified breads with raw YSP flour; however, both attributes were eliminated only in the case of 10% roasted YSP addition. Therefore, breads with 10% roasted YSP could be appealing to a wider diet-conscious consumer group. Finally, inclusion of YSP flours into wheat bread formulation enhances the nutritional quality of the product by increasing the protein and the total dietary fiber content, as well as reducing starch digestibility rates as monitored by *in vitro* testing. Overall, substitution of wheat flour with roasted YSP flour at 10% level was the most successful treatment, since it did not adversely affect dough rheology, bread texture, and staling kinetics, as well as the product sensory attributes. It is obvious that flours from roasted YSP can be effectively incorporated into wheat bread formulations in order to improve quality and nutritional characteristics of the final product when introduced at relatively low levels of fortification. 

## Figures and Tables

**Table 1:** Effect of flour from raw and roasted yellow split pea on gelatinization properties of wheat flour slurries (flour: water 30:70 *w/w*) derived from differential scanning calorimetry (DSC).

Sample Symbol	Sample Formulation	Gelatinization Properties		
		Onset Temperature, T_o_ (°C)	Peak Temperature, T_p_ (°C)	Apparent Enthalpy, ΔH_gel_ (mJ/mg Starch)
WF (CON)	100% wheat flour	55.45 a ^1^	63.76 a	7.19 a
YSPF	100% raw yellow split pea flour	71.18 b	78.31 b	6.75 a
RYSPF	100% roasted yellow split pea flour	71.22 b	78.66 b	6.93 a
YSP10	Mixture of 10% YSPF and 90% WF	55.86 a	64.05 a	6.74 a
RYSP10	Mixture of 10% RYSPF and 90% WF	55.90 a	64.01 a	6.81 a
RYSP15	Mixture of 15% RYSPF and 85% WF	n.d. ^2^	n.d.	n.d.
RYSP20	Mixture of 20% RYSPF and 80% WF	56.30 a	64.33 a	7.10 a

^1^ Mean values with a same letter in the same column are not significantly different according to Tukey’s test (*p* > 0.05). ^2^ n.d.: not determined.

**Table 2:** Empirical dough properties of yellow split pea fortified wheat flour as determined by farinography and extensograghy.

	CON ^1^	YSP10	RYSP10	RYSP15	RYSP20
**Farinograph**					
Water absorption (%)	58.5 a ^2^	60.5 ab	61.5 ab	61.5 ab	62.5 b
Development time (min)	2.3 ab	1.8 a	3.0 bc	3.2 c	4.2 d
Stability (min)	10.2 b	10.4 b	12.1 b	7.5 a	6.1 a
**Extensograph**					
**Stretch energy: Area (cm^2^)**					
45 min	96.9 b,B ^3^	118.7 c,B	105.9 bc,A	96.8 b,A	66.9 a,A
90 min	66.8 a,A	116.3 c, AB	109.6 bc,A	94.9 b,A	66.0 a,A
135 min	72.6 ab,A	104.6 c, A	110.9 c,A	93.0 bc,A	55.6 a,A
**Resistance to extension at 50 mm (R50, BU)**					
45 min	410 ab,A	470 b,A	400 ab,A	415 ab,A	340 a,A
90 min	580 c,B	625 c,B	440 b,A	415 b,A	310 a,A
135 min	560 b,B	555 b,AB	450 b A	440 b,A	300 a,A
**Extensibility (E, mm)**					
45 min	142 b,B	137 ab,A	152 b,A	139 ab,A	127 a,A
90 min	90 a,A	124 b,A	152 c,A	146 bc,A	124 b,A
135 min	94 a,A	130 b,A	154 c,A	137 bc,A	132 bc,A
**R50/E**					
45 min	2.9 a,A	3.4 a,A	2.6 a,A	2.9 a,A	2.7 a,A
90 min	6.5 c,B	5.1 b,B	2.9 a,A	2.8 a,A	2.5 a,A
135 min	6.0 c,B	4.3 b,AB	3.0 ab,A	3.2 ab,A	2.3 a,A

^1^ Notation of samples as in Table 1. ^2^ Mean values with the same lowercase letter in the same row are not significantly different according to Tukey’s test (*p* > 0.05). ^3^ Mean values with the same uppercase letter in the same column, for the same rheological parameter, are not significantly different according to Tukey’s test (*p* > 0.05).

**Table 3:** Effect of flour from raw and roasted yellow split pea on rheological parameters of wheat flour doughs derived from frequency sweep and creep-recovery test.

Rheological Parameters	CON ^1^	YSP10	RYSP10	RYSP15	RYSP20
**Frequency Sweep Test**					
Storage modulus (G′) at 5.37 Hz (kPa)	12.6 ab ^2^	8.4 a	12.5 ab	15.1 ab	17.2 b
Loss modulus (G″) at 5.37 Hz (kPa)	4.6 ab	2.8 a	4.0 ab	5.2 b	5.4 b
Damping factor (tan δ) at 5.37 Hz	0.36 a	0.35 a	0.33 a	0.36 a	0.32 a
Complex viscosity (η*) at 5.37 Hz (kPa⋅s)	0.40 ab	0.26 a	0.39 ab	0.48 ab	0.53 b
**Creep-Recovery Test**					
Maximum creep strain %	1.85 b	1.23 a	1.40 ab	1.30 ab	1.26 a
**Burgers Model Fitting**					
Max. creep compliance, J_max_ (1/Pa) × 10^4^	15.68 b	9.31 a	11.61 ab	9.92 a	8.89 a
**Creep phase**					
Instantaneous compliance, J_o_ (1/Pa) × 10^4^	3.49 a	2.45 a	2.81 a	2.60 a	2.53 a
Viscoelastic compliance, J_m_ (1/Pa) × 10^4^	5.69 b	3.22 a	4.01 ab	3.13 a	3.06 a
Zero shear viscosity, η_o_ (Pa∙s) × 10^−6^	0.20 a	0.32 ab	0.24 ab	0.29 ab	0.34 b
**Recovery phase**					
Instantaneous compliance, J_o_ (1/Pa) × 10^4^	4.24 a	3.94 a	4.23 a	4.02 a	3.23 a
Viscoelastic compliance, J_m_ (1/Pa) × 10^4^	2.71 b	2.36 ab	2.61 b	2.40 ab	1.71 a
Mean retardation time, λ (s)	62.1 a	65.5 a	64.1 a	61.5 a	55.1 a
Relative elastic portion of J_max_, J_e_/_Jmax_ (%)	61.8 a	64.3 a	57.0 a	59.2 a	55.3 a

^1^ Notation of samples as in Table 1. ^2^ Mean values with the same letter in the same row are not significantly different according to Tukey’s test (*p* > 0.05).

**Table 4:** Appearance parameters of wheat-based breads fortified with yellow split pea flours.

	CON ^1^	YSP10	RYSP10	RYSP15	RYSP20
Loaf-specific volume (mL/g)	2.81 c ^2^	2.67 bc	2.92 c	2.41 ab	2.30 a
**Bread crust color**					
L*	60.7 b	48.2 ab	52.3 b	45.7 a	44.3 a
a*	10.1 a	13.3 bc	13.0 b	14.1b c	14.9 c
b*	32.5 c	28.5 ab	31.2 bc	26.6 a	26.4 a
c*	34.0 b	31.5 ab	33.8 b	30.5 a	30.2 a
h_ab_ (^o^)	72.8 d	64.9 bc	67.2 c	61.9 ab	60.2 a
**Bread crumb color**					
L*	68.4 a	67.8 a	68.1 a	66.2 ab	64.9 b
a*	−1.5 a	−1.7 a	−1.7 a	−1.9 a	−1.0 b
b*	15.8 a	16.2 ab	16.8 ab	18.6 ab	20.1 b
c*	15.9 a	16.3 ab	16.9 ab	18.6 ab	20.7 b
h_ab_ (^o^)	95.7 b	96.1 b	95.7 b	95.8 b	92.9 a

^1^ Notation of samples as in Table 1. ^2^ Mean values with the same letter in the same row are not significantly different according to Tukey’s test (*p* > 0.05).

**Table 5:** Crumb and crust texture characteristics assessed by TPA and puncture test, respectively, and moisture contents of fresh wheat-based breads fortified with yellow split pea flours, and their change rate during product storage at 25 °C.

	CON ^3^	YSP10	RYSP10	RYSP15	RYSP20
**Crumb**					
Hardness (N) ^1^	10.59 a ^4^	11.91 ab	8.92 a	14.88 bc	17.65 c
Hardening rate (N⋅d^−1^) ^2^	3.80 ab	2.56 a	2.45 a	3.40 ab	4.19 b
Cohesiveness ^1^	0.68 a	0.66 a	0.69 a	0.67 a	0.66 a
Cohesiveness loss rate (d^−1^) ^2^	0.06 a	0.06 a	0.06 a	0.07 a	0.06 a
Resilience ^1^	0.38 b	0.35 ab	0.37 ab	0.36 ab	0.33 a
Resilience loss rate (d^−1^) ^2^	0.05 a	0.04 a	0.04 a	0.05 a	0.04 a
Springiness ^1^	0.92 a	0.91 a	0.93 a	0.90 a	0.90 a
Springiness loss rate (d^−1^) ^2^	0.02 a	0.02 a	0.02 a	0.02 a	0.03 a
Chewiness ^1^ (N)	6.60 a	6.47 a	5.84 a	7.90 a	10.50 b
Chewiness increase rate (N⋅d^−1^) ^2^	0.49 b	0.28 ab	0.28 ab	0.19 ab	0.10 a
Moisture content ^1^ (%)	43.53 a	43.76 a	43.92 a	44.29 a	44.32 a
Moisture loss rate (% ⋅ d^−1^) ^2^	0.44 a	0.36 ab	0.47 a	0.30 ab	0.18 b
**Crust**					
Hardness (N) ^1^	10.50 a	11.18 a	12.25 a	12.11 a	10.45 a
Softening rate (N⋅d^−1^) ^2^	1.06 b	1.80 a	1.51 ab	1.85 a	1.68 ab
Moisture content (%) ^1^	17.11 a	19.16 b	19.41 bc	20.71 bc	21.58 d
Moisture gain rate (% d^−1^) ^2^	1.99 a	1.86 a	1.34 a	2.00 a	1.99 a

^1^ Textural parameters and moisture contents evaluated after 2 h of bread storage (0 day).^2^ Calculated from the slope of the linear regression model fitted to the data of the textural parameter or moisture values versus storage time. ^3^ Notation of samples as in Table 1. ^4^ Mean values with the same letter in the same row are not significantly different according to Tukey’s test (*p* > 0.05).

**Table 6:** Starch retrogradation parameters of crumbs of wheat breads fortified with yellow split pea flour as evaluated by DSC analysis; breads were stored at 25 °C.

Starch Retrogradation Parameters	CON ^4^	YSP10	RYSP10	RYSP20
Onset temperature, T_o_^ret^ (°C) ^1^	45.37 a ^5^	45.21 a	45.98 a	45.43 a
Peak temperature, T_p_^ret^ (°C) ^1^	55.09 a	54.11 a	55.22 a	56.32 a
Apparent melting enthalpy, ΔH_ret_ (mJ/mg starch) ^1^	2.51 a	2.21 a	2.36 a	3.08 b
ΔH_ret_ increase rate (mJ/mg starch/d) ^2^	0.53 a	0.48 a	0.52 a	0.65 b
RI (%) ^3^	35.0 a	34.7 a	35.6 a	43.3 b

^1^ T_o_^ret^, T_p_^ret^, ΔH_ret_: parameters of melting of the retrograded amylopectin at the 4th day of bread storage. ^2^ ΔH_ret_ increase rate: calculated from the slope of the linear regression model fitted to the data of the apparent melting enthalpy of the retrograded amylopectin, ΔH_ret_, versus storage time. ^3^ RI (%): retrogradation index calculated at the 4th day of bread storage. ^4^ Notation of samples as in Table 1. ^5^ Mean values with the same letter in the same row are not significantly different according to Tukey’s test (*p* > 0.05).

**Table 7:** Secondary structure of proteins and starch chain ordering of wheat and yellow split pea flours and crumbs of wheat breads fortified with yellow split pea flour as evaluated by FTIR spectroscopy; breads were stored at 25 °C.

Flours					
		Samples ^1^			
Secondary Structure (%)		WF		YSPF	RYSPF
β-sheet		41.5 ± 3.49 a ^2^		47.80 ± 0.18 a	45.36 ± 3.81 a
random		3.51 ± 0.77 a		13.31 ± 0.15 b	15.42 ± 0.21 b
α-helix		28.21 ± 1.22 b		17.66 ± 0.21 a	17.27 ± 2.10 a
β-turn		17.19 ± 0.22 b		14.65 ± 2.40 a	15.22 ± 2.40 ab
**Ratio of ordered to amorphous starch, R** **_1047/1022_**		0.455 ± 0.006 b		0.455 ± 0.003 b	0.432 ± 0.000 a
**Bread Crumb**					
		**Storage Time**			
**Samples** ^1^	**Secondary Structure (%)**	**0 Day**		**4 Day**	
**CON**	β-sheet	26.12 ± 2.33 a ^2^		25.87 ± 0.58 a	
	random	24.54 ± 0.39 a		26.79 ± 0.78 b	
	α-helix	29.08 ± 4.15 a		26.81 ± 1.67 a	
	β-turn	10.69 ± 2.83 a		11.68 ± 0.77 a	
**YSP10**	β-sheet	27.93 ± 0.98 a		26.12 ± 0.56 a	
	random	25.30 ± 4.70 a		31.69 ± 2.49 a	
	α-helix	25.89 ± 2.89 b		19.07 ± 1.21 a	
	β-turn	11.67 ± 0.70 a		13.64 ± 0.47 b	
**RYSP10**	β-sheet	25.80 ± 1.44 a		29.28 ± 0.21 b	
	random	28.52 ± 5.93 a		21.98 ± 2.10 a	
	α-helix	25.17 ± 6.66 a		28.66 ± 2.40 a	
	β-turn	11.33 ± 1.31 a		11.07 ± 0.18 a	
**RYSP20**	β-sheet	52.20 ± 3.27 a		53.20 ± 1.63 a	
	random	3.86 ± 1.05 a		3.30 ± 0.46 a	
	α-helix	10.90 ± 0.57 b		5.72 ± 1.97a	
	β-turn	26.04 ± 0.75 a		29.06 ± 1.50 b	
		**Ratio of Ordered to Amorphous Starch,** **R_1047/1022_**			
		**Storage Time**			
**Samples ^1^**		**0 Day**		**4 Day**	
**CON**		0.335 ± 0.003 a ^2^		0.340 ± 0.002 a	
**YSP10**		0.338 ± 0.001 a		0.343 ± 0.001 a	
**RYSP10**		0.337 ± 0.003 a		0.342 ± 0.003 a	
**RYSP20**		1.104 ± 0.003 a		1.188 ± 0.005 b	
**Samples ^1^**		**CON**	**YSP10**	**RYSP10**	**RYSP20**
**Rate of R_1047/1022_ increase (day^−1^) ^3^**		0.0011 a ^2^	0.0012 a	0.0013 a	0.0198b

^1^ Notation of samples as in Table 1. ^2^ Mean values with a same letter in the same row are not significantly different according to Tuckey’s test (*p* > 0.05). ^3^ Calculated from the slope of the linear regression model fitted to the data of the R_1047/1022_ values versus storage time.

**Table 8:** Proximate composition and *in-vitro* starch digestibility of breads fortified with yellow split pea flours.

Samples	Protein	TDF ^2^	Carbohydrates	Fat	TDS ^2^	RS ^2^	AUC ^6^
	g/100 g Bread				g/100 g b.c. ^5^		(g Glucose /g TDS)·min
CON ^1^	8.0	2.0	52.1	1.1	36.3 a ^3^	0.55 a	97.2 b
YSP10	9.1	3.0	49.6	1.1	36.6 a	0.93 ab	88.2 b
RYSP10	9.1	3.0	49.9	1.1	35.1 a	0.82 ab	93.3 b
RYSP15	9.5	3.4	47.6	1.1	n.d. ^4^	n.d.	n.d.
RYSP20	10.0	3.8	45.9	1.1	34.9 a	1.52 c	78.4 a

^1^ Notation of samples as in Table 1. ^2^ TDF: total dietary fiber; TDS: total digestible starch; RS: resistant starch. ^3^ n.d.: mean values with a same letter in the same column are not significantly different according to Tukey’s test (*p* > 0.05). ^4^ n.d.: not determined. ^5^ b.c.: bread crumb. ^6^ AUC: area under the curve of released glucose over 300 min of *in vitro* starch digestion.

## Supplementary Materials

The following are available online at https://www.mdpi.com/article/10.3390/foods10081832/s1. Figure S1. Cumulative distribution of particle size of wheat, and raw and roasted yellow split pea flours. Notation of samples as in Table 1; Figure S2. Representative DSC thermograms of starch gelatinization apparent enthalpy (ΔH_gel_) of wheat flours, yellow split pea flours, and their mixtures. Notation of samples as in Table 1; Table S1. Effect of flour from raw and roasted yellow split pea on gelatinization properties of wheat flour slurries (flour: water 30: 70 w/w) derived from differential scanning calorimetry (DSC); Table S2. Empirical dough properties of yellow split pea fortified wheat flour as determined by farinoghraphy and extensograghy; Table S3. Effect of flour from raw and roasted yellow split pea on rheological parameters of wheat flour doughs derived from frequency sweep and creep-recovery test; Table S4. Appearance parameters of wheat-based breads fortified with yellow split pea flours; Table S5. Crumb and crust texture characteristics assessed by TPA and puncture test, respectively and moisture contents of fresh wheat-based breads fortified with yellow split pea flours, and their change rate during product storage at 25 °C; Table S6. Starch retrogradation parameters of crumbs of wheat breads fortified with yellow split pea flour as evaluated by DSC analysis; breads were stored at 25°C; Table S7. Proximate composition and *in vitro* starch digestibility of breads fortified with yellow split pea flours.
