# Supplementary material for: Impact of Roasted Yellow Split Pea Flour on Dough Rheology and Quality of Fortified Wheat Breads
Source: Foods. 2021 Aug 7;10(8):1832. doi: 10.3390/foods10081832 (PMC8391420; doi:10.3390/foods10081832)
Supplement: Supplementary file 1 [file foods-10-01832-s001.zip › Supplementary - foods - 1289337.pdf]

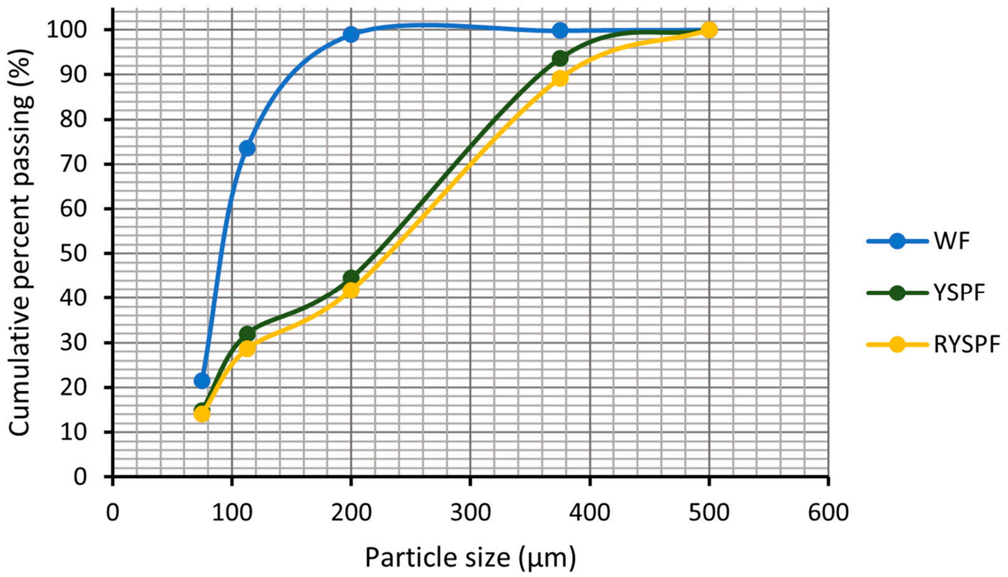

Figure S1. Cumulative distribution of particle size of wheat, and raw and roasted yellow split pea flours. Notation of samples as in Table 1.

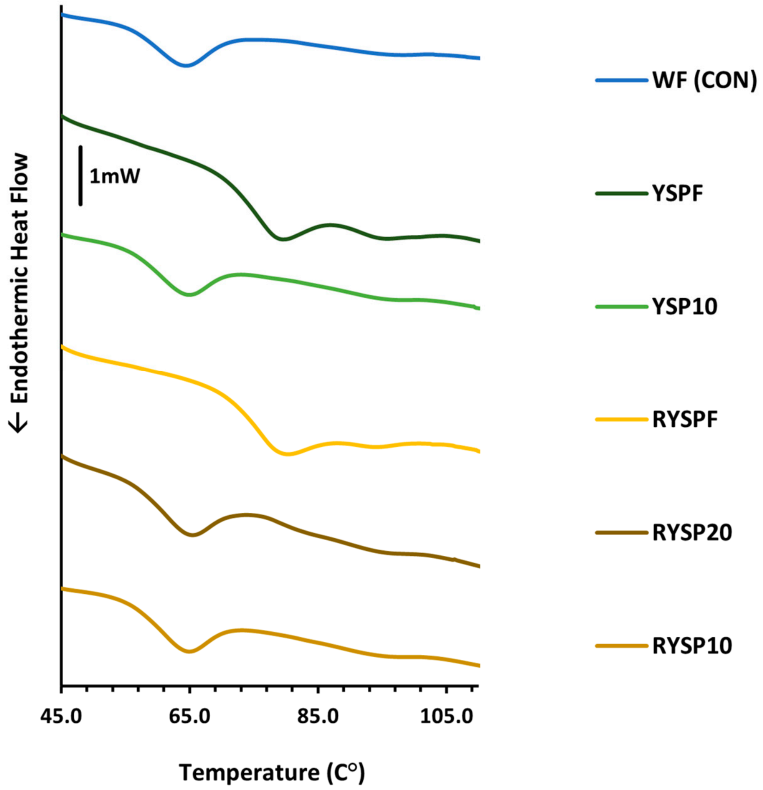

Figure S2. Representative DSC thermograms of starch gelatinization apparent enthalpy (ΔH_{gel}) of wheat flours, yellow split pea flours, and their mixtures. Notation of samples as in Table 1.

Table S1. Effect of flour from raw and roasted yellow split pea on gelatinization properties of wheat flour slurries (flour: water 30: 70 w/w) derived from differential scanning calorimetry (DSC).

Sample symbol	Sample formulation	Gelatinization properties		
		**Onset temperature, T_o (°C)**	**Peak temperature, T_p (°C)**	**Apparent enthalpy, ΔH_{gel} (mJ/mg starch)**
WF (CON)	100% Wheat flour	55.45 ± 0.31 a [1]	63.76 ± 0.06 a	7.19 ± 0.50 a
YSPF	100% Raw yellow split pea flour	71.18 ± 0.18 b	78.31 ± 0.20 b	6.75 ± 0.73 a
RYSPF	100% Roasted yellow split pea flour	71.22 ± 0.04 b	78.66 ± 0.37 b	6.93 ± 0.78 a
YSP10	Mixture of 10% YSPF and 90% WF	55.86 ± 0.16 a	64.05 ± 0.01a	6.74 ± 0.29 a
RYSP10	Mixture of 10% RYSPF and 90% WF	55.90 ± 0.22 a	64.01 ± 0.20a	6.81 ± 0.30a
RYSP15	Mixture of 15% RYSPF and 85% WF	n.d. [2]	n.d.	n.d.
RYSP20	Mixture of 20% RYSPF and 80% WF	56.30 ± 0.25 a	64.33 ± 0.66 a	7.10 ± 0.66a

[1] Mean values with a same letter in the same column are not significantly different according to Tukey's test ($p > 0.05$).

[2] n.d.: not determined.

Table S2. Empirical dough properties of yellow split pea fortified wheat flour as determined by farinoghraphy and extensograghy.

	CON [1]	YSP10	RYSP10	RYSP15	RYSP20
**Farinograph**					
Water absorption (%)	58.5 ± 0.5 a [2]	60.5 ± 1.0 ab	61.5 ± 1.0 ab	61.5 ± 1.0 ab	62.5 ± 1.0 b
Development time (min)	2.3 ± 0.3 ab	1.8 + 0.4 a	3.0 ± 0.2 bc	3.2 ± 0.3 c	4.2 ± 0.2 d
Stability(min)	10.2 ± 0.6 b	10.4 ± 0.7 b	12.1 ± 1.5 b	7.5 ± 0.5 a	6.1 ± 0.3 a
**Extensograph**					
**Stretch energy: Area (cm2)**					
45 min	96.9 ± 8.8 b, B [3]	118.7 ± 7.8 c, B	105.9 ± 5.9 bc, A	96.8 ± 6 b, A	66.9 ± 11.8 a, A
90 min	66.8 ± 5.5 a, A	116.3 ± 5.7 c, AB	109.6 ± 10.8 bc, A	94.9 ± 2 b, A	66.0 ± 10.6 a, A
135 min	72.6 ± 6.8 ab, A	104.6 ± 10.3 c, A	110.9 ± 5.9 c, A	93.0 ± 3 bc, A	55.6 ± 10.5 a, A
**Resistance to extension at 50mm (R50, BU)**					
45 min	410 ± 30 ab, A	470 ± 60 b, A	400 ± 30ab, A	415 ± 10 ab, A	340 ± 30 a, A
90 min	580 ± 10 c, B	625 ± 20 c, B	440 ± 30 b, A	415 ± 10 b, A	310 ± 40 a, A
135 min	560 ± 25 b, B	555 ± 50 b, AB	450 ± 60 b, A	440 ± 30 b, A	300 ± 30 a, A
**Extensibility (E, mm)**					
45 min	142 ±4 b, B	137 ± 8 ab, A	152 ± 7 b, A	139 ± 2 ab, A	127 ± 10 a, A
90 min	90 ± 10 a, A	124 ± 4 b, A	152 ± 4 c, A	146 ± 7 bc, A	124 ± 20 b, A
135 min	94 ± 6 a, A	130 ± 8 b, A	154 ± 13 c, A	137 ± 2 bc, A	132 ± 15 bc, A
**R50/E**					
45 min	2.9 ± 0.3 a, A	3.4 ± 0.6 a, A	2.6 ± 0.3 a, A	2.9 ± 0.1 a, A	2.7 ± 0.2 a, A
90 min	6.5 ± 0.7 c, B	5.1 ± 0.1 b, B	2.9 ± 0.3 a, A	2.8 ± 0.2 a, A	2.5 ± 0.2 a, A
135 min	6.0 ± 0.5 c, B	4.3 ± 1.1 b, AB	3.0 ± 0.6 ab, A	3.2 ± 0.2 ab, A	2.3 ± 0.2 a, A

[1] Notation of samples as in Table 1.

[2] Mean values with the same lowercase letter in the same row are not significantly different according to Tukey's test ($p > 0.05$).

[3] Mean values with the same uppercase letter in the same column, for the same rheological parameter, are not significantly different according to Tukey's test ($p > 0.05$).

Table S3. Effect of flour from raw and roasted yellow split pea on rheological parameters of wheat flour doughs derived from frequency sweep and creep-recovery test.

Rheological parameters	CON [1]	YSP10	RYSP10	RYSP15	RYSP20
**Frequency sweep test**					
Storage modulus (G′) at 5.37 Hz (kPa)	12.6 ± 1.1 ab [2]	8.4 ± 1.7a	12.5 ± 3.5 ab	15.1 ± 1.1ab	17.2 ± 5.0 b
Loss modulus (G″) at 5.37 Hz (kPa)	4.6 ± 0.5 ab	2.8 ± 0.4 a	4.0 ± 1.0 ab	5.2 ± 0.2 b	5.4 ± 1.1 b
Damping factor (tanδ) at 5.37 Hz	0.36 ± 0.01 a	0.35 ± 0.06 a	0.33 ± 0.05 a	0.36 ± 0.01a	0.32 ± 0.04 a
Complex viscosity (η*) at 5.37 Hz (kPa·s)	0.40 ± 0.03ab	0.26 ± 0.05 a	0.39 ± 0.10 ab	0.48 ± 0.03ab	0.53 ± 0.15 b
**Creep-recovery test**					
Maximum creep strain %	1.85 ± 0.41 b	1.23 ± 0.07a	1.40 ± 0.13 ab	1.30 ± 0.14 ab	1.26 ± 0.12 a
**Burgers model fitting**					
Max. Creep Compliance, J_{max} (1/Pa) x 10^4	15.68 ± 3.21 b	9.31 ± 0.42 a	11.61 ± 1.50 ab	9.92 ± 2.21 a	8.89 ± 0.45 a
**Creep phase**					
Instantaneous Compliance, J_o (1/Pa) x 10^4	3.49 ± 0.82 a	2.45 ± 0.15 a	2.81 ± 0.25 a	2.60 ± 0.28 a	2.53 ± 0.25 a
Viscoelastic Compliance, J_m (1/Pa) x 10^4	5.69 ± 1.21 b	3.22 ± 0.35 a	4.01 ± 0.64 ab	3.13 ± 0.72 a	3.06 ± 0.27 a
Zero Shear Viscosity, η_o (Pa·s) x 10^{-6}	0.20 ± 0.05 a	0.32 ± 0.06 ab	0.24 ± 0.03 ab	0.29 ± 0.09ab	0.34 ± 0.01 b
**Recovery phase**					
Instantaneous Compliance, J_o (1/Pa) x 10^4	4.24 ± 0.52 a	3.94 ± 0.5 a	4.23 ± 0.41 a	4.02 ± 0.19 a	3.23 ± 0.60 a
Viscoelastic Compliance, J_m (1/Pa) x 10^4	2.71 ± 0.35 b	2.36 ± 0.04 ab	2.61 ± 0.24 b	2.40 ± 0.06 ab	1.71 ± 0.50 a
Mean Retardation Time, λ (s)	62.1 ± 10.1 a	65.5 ± 7.9 a	64.1 ± 5.3a	61.5 ± 0.7a	55.1 ± 1.7 a
Relative elastic portion of J_{max}, J_e/J_{max} (%)	61.8 ± 5.5 a	64.3 ± 4.7 a	57.0 ± 4.4 a	59.2 ± 4.2 a	55.3 ± 7.0 a

[1] Notation of samples as in Table 1.

[2] Mean values with the same letter in the same row are not significantly different according to Tukey's test ($p > 0.05$).

Table S4. Appearance parameters of wheat-based breads fortified with yellow split pea flours.

	**CON** [1]	**YSP10**	**RYSP10**	**RYSP15**	**RYSP20**
Loaf specific volume (ml/g)	2.81 ± 0.13 c [2]	2.67 ± 0.13 bc	2.92 ± 0.16 c	2.41 ± 0.09 ab	2.30 ± 0.10 a
**Bread crust color**					
L*	60.7 ± 2.8 b	48.2 ± 1.4 ab	52.3 ± 0.8 b	45.7 ± 1.1 a	44.3 ± 1.0 a
a*	10.1 ± 1.2 a	13.3 ± 0.4 bc	13.0 ± 0.4 b	14.1 ± 0.4 bc	14.9 ± 0.4 c
b*	32.5 ± 2.3 c	28.5 ± 0.6 ab	31.2 ± 0.5 bc	26.6 ± 0.6 a	26.4 ± 0.8 a
C*	34.0 ± 2.3 b	31.5 ± 0.5 ab	33.8 ± 0.5 b	30.5 ± 0.7 a	30.2 ± 0.4a
h_{ab} (°)	72.8 ± 1.9 d	64.9 ± 0.9 bc	67.2 ± 0.5 c	61.9 ± 1.1 ab	60.2 ± 1.0 a
**Bread crumb color**					
L*	68.4 ± 1.0 a	67.8 ± 0.5 a	68.1 ± 0.6 a	66.2 ± 1.5 ab	64.9 ± 1.1 b
a*	-1.5 ± 0.14 a	-1.7 ± 0.1 a	-1.7 ± 0.1 a	-1.9 ± 0.1 a	-1.0 ± 0.3 b
b*	15.8 ± 3.5 a	16.2 ± 0.5 ab	16.8 ± 0.2 ab	18.6 ± 0.3 ab	20.1 ± 1.2 b
C*	15.9 ± 3.5 a	16.3 ± 0.5 ab	16.9 ± 0.3ab	18.6 ± 0.3 ab	20.7 ± 1.3 b
h_{ab} (°)	95.7 ± 0.3 b	96.1 ± 0.2 b	95.7 ± 0.3 b	95.8 ± 0.2b	92.9 ± 0.7 a

[1] Notation of samples as in Table 1.

[2] Mean values with the same letter in the same row are not significantly different according to Tukey's test ($p > 0.05$).

Table S5. Crumb and crust texture characteristics assessed by TPA and puncture test, respectively and moisture contents of fresh wheat-based breads fortified with yellow split pea flours, and their change rate during product storage at 25 °C.

	CON [3]	YSP10	RYSP10	RYSP15	RYSP20
**Crumb**					
Hardness (N) [1]	10.59 ± 0.5 a [4]	11.91 ± 1.34 ab	8.92 ± 1.73 a	14.88 ± 2.42 bc	17.65 ± 1.60 c
Hardening rate ($N \cdot d^{-1}$) [2]	3.80 ± 0.30 ab	2.56 ± 0.51 a	2.45 ± 0.45 a	3.40 ± 0.49 ab	4.19 ± 0.92 b
Cohesiveness [1]	0.68 ± 0.01 a	0.66 ± 0.03 a	0.69± 0.02 a	0.67 ± 0.02 a	0.66 ± 0.01 a
Cohesiveness loss rate (d^{-1}) [2]	0.06 ± 0.01 a	0.06 ± 0.00a	0.06 ± 0.01 a	0.07 ± 0.00 a	0.06 ± 0.00 a
Resilience [1]	0.38 ± 0.01 b	0.35 ± 0.02 ab	0.37 ± 0.01 ab	0.36 ± 0.02 ab	0.33 ± 0.01 a
Resilience loss rate (d^{-1}) [2]	0.05 ± 0.00 a	0.04 ± 0.00 a	0.04 ± 0.01 a	0.05 ± 0.01 a	0.04 ± 0.00 a
Springiness [1]	0.92 ± 0.01 a	0.91 ± 0.02 a	0.93 ± 0.02 a	0.90 ± 0.01 a	0.90 ± 0.01 a
Springiness loss rate (d^{-1}) [2]	0.02 ± 0.01 a	0.02 ± 0.01 a	0.02 ± 0.01 a	0.02 ± 0.01 a	0.03 ± 0.01 a
Chewiness [1] (N)	6.60 ± 0.25 a	6.47 ±0.79 a	5.84 ± 1.06 a	7.90 ± 0.75 a	10.50 ± 1.18 b
Chewiness increase rate ($N \cdot d^{-1}$) [2]	0.49 ± 0.09 b	0.28 ± 0.06 ab	0.28 ± 0.11 ab	0.19 ± 0.05 ab	0.10 ± 0.03a
Moisture content [1] (%)	43.53 ± 0.24 a	43.76 ± 0.21 a	43.92 ± 0.23 a	44.29 ± 0.23 a	44.32 ± 0.58 a
Moisture loss rate ($\% \cdot d^{-1}$) [2]	0.44 ± 0.07 a	0.36 ± 0.12 ab	0.47 ± 0.06 a	0.30 ± 0.08 ab	0.18 ± 0.04 b
**Crust**					
Hardness (N) [1]	10.50 ± 0.17 a	11.18 ± 1.53 a	12.25 ± 0.21 a	12.11 ± 1.53 a	10.45 ± 1.90 a
Softening rate ($N \cdot d^{-1}$) [2]	1.06 ± 0.11 b	1.80 ± 0.25 a	1.51 ± 0.22 ab	1.85 ± 0.28 a	1.68 ± 0.34 ab
Moisture content (%) [1]	17.11 ± 0.41 a	19.16 ± 0.89 b	19.41 ± 0.61 bc	20.71 ± 0.11 bc	21.58 ± 0.22 d
Moisture gain rate ($\% \cdot d^{-1}$) [2]	1.99 ± 0.35 a	1.86 ± 0.16 a	1.34 ± 0.36 a	2.00 ± 0.25 a	1.99 ± 0.10a

[1] Textural parameters and moisture contents evaluated after 2 h of bread storage (0 day).

[2] Calculated from the slope of the linear regression model fitted to the data of the textural parameter or moisture values versus storage time.

[3] Notation of samples as in Table 1.

[4] Mean values with the same letter in the same row are not significantly different according to Tukey's test ($p > 0.05$).

Table S6. Starch retrogradation parameters of crumbs of wheat breads fortified with yellow split pea flour as evaluated by DSC analysis; breads were stored at 25°C.

Starch retrogradation parameters	CON [6]	YSP10	RYSP10	RYSP20
Onset temperature, T_o^{ret} (°C) [1]	45.37 ± 0.87 a [7]	45.21 ± 0.47 a	45.98 ± 0.51 a	45.43 ± 1.09a
Peak temperature, T_p^{ret} (°C) [1]	55.09 ± 1.17 a	54.11 ± 0.09 a	55.22 ± 0.61 a	56.32 ± 0.39a
Apparent melting enthalpy, ΔH_{ret} (mJ/mg starch) [1]	2.51 ± 0.11 a	2.21 ± 0.25 a	2.36 ± 0.03 a	3.08 ± 0.20 b
ΔH_{ret} increase rate (mJ/mg starch/d) [2]	0.53 ± 0.03 a	0.48 ± 0.05 a	0.51 ± 0.02 a	0.65 ± 0.05 b
RI (%) [3]	35.0 a	34.7 a	35.6 a	43.3 b

[1] T_o^{ret}, T_p^{ret}, ΔH_{ret}: parameters of melting of the retrograded amylopectin at the 4th day of bread storage.

[2] ΔH_{ret} increase rate: calculated from the slope of the linear regression model fitted to the data of the apparent melting enthalpy of the retrograded amylopectin, ΔH_{ret}, versus storage time.

[3] RI (%): retrogradation index calculated at the 4th day of bread storage.

[4] Notation of samples as in Table 1.

[5] Mean values with the same letter in the same row are not significantly different according to Tukey's test ($p > 0.05$).

Table S7. Proximate composition and *in vitro* starch digestibility of breads fortified with yellow split pea flours.

	Protein	TDF [2]	Carbohydrates	Fat	TDS [2]	RS [5]	AUC [6]
	g/100g bread				g/100g b.c. [4]		(g glucose /g TDS)·min
CON [1]	8.0	2.0	52.1	1.1	36.3 ± 1.5a [3]	0.55 ± 0.15 a	97.2 ± 6.2 b
YSP10	9.1	3.0	49.6	1.1	36.6 ± 1.1 a	0.93 ± 0.10 ± ab	88.2 ± 3.5 b
RYSP10	9.1	3.0	49.9	1.1	35.1 ± 1.2 a	0.82 ± 0. 15 ab	93.3 ± 12.0 b
RYSP15	9.5	3.4	47.6	1.1	n.d. [4]	n.d.	n.d.
RYSP20	10.0	3.8	45.9	1.1	34.9 ± 1.0 a	1.52 ± 0.32c	78.4 ± 5.0 a

[1] Notation of samples as in Table 1.

[2] TDF: Total Dietary Fiber; TDS: Total Digestible Starch; RS: Resistant starch.

[3] Mean values with the same letter in the same column are not significantly different according to Tukey's test ($p > 0.05$).

[4] n.d.: not determined.

[5] b.c.: bread crumb.

[6] AUC: Area under the curve of released glucose over 300 min of *in vitro* starch digestion.
